# Significance of serglycin and its binding partners in autocrine promotion of metastasis in esophageal cancer

**DOI:** 10.7150/thno.49547

**Published:** 2021-01-01

**Authors:** Yun Zhu, Alfred K.Y. Lam, Daisy K.Y. Shum, Di Cui, Jun Zhang, Dong Dong Yan, Bin Li, Wen Wen Xu, Nikki P.Y. Lee, Kin Tak Chan, Simon Law, Sai Wah Tsao, Annie L.M. Cheung

**Affiliations:** 1 School of Biomedical Sciences, Li Ka Shing Faculty of Medicine, University of Hong Kong, Hong Kong SAR, China; 2 Department of Pathology, Griffith Medical School, Queensland, Gold Coast, QLD, Australia; 3 MOE Key Laboratory of Tumor Molecular Biology and Key Laboratory of Functional Protein Research of Guangdong Higher Education Institutes, Institute of Life and Health Engineering, Jinan University, Guangzhou, China; 4 MOE Key Laboratory of Tumor Molecular Biology and Guangdong Provincial Key Laboratory of Bioengineering Medicine, National Engineering Research Center of Genetic Medicine, Institute of Biomedicine, College of Life Science and Technology, Jinan University, Guangzhou, China; 5 Department of Surgery, Li Ka Shing Faculty of Medicine, University of Hong Kong, Hong Kong SAR, China

**Keywords:** Serglycin, midkine, esophageal squamous cell carcinoma, metastasis, biomarker

## Abstract

**Rationale**: Little is known about the roles of proteoglycans in esophageal cancer. This study aims to investigate the roles and mechanisms of serglycin (SRGN) proteoglycan in promoting metastasis of esophageal squamous cell carcinoma (ESCC).

**Methods**: Reverse phase protein array analysis was used to identify activated signaling pathways in *SRGN*-overexpressing cells. Chemokine array was used to identify differentially secreted factors from *SRGN*-overexpressing cells. Binding between SRGN and potential interacting partners was evaluated using proximity ligation assay and co-immunoprecipitation. The glycosaminoglycan (GAG) chains of SRGN were characterized using fluorophore-assisted carbohydrate electrophoresis. Tissue microarray and serum samples were used to determine the correlation of SRGN expression with clinicopathological parameters and patient survival.

**Results**: *In vitro* and *in vivo* experiments showed that SRGN promoted invasion and metastasis in ESCC via activating ERK pathway, stabilizing c-Myc and upregulating the secretion of matrix metalloproteinases. *SRGN*-knockdown suppressed tumorigenic hallmarks. These SRGN-elicited functions were carried out in an autocrine manner by inducing the secretion of midkine (MDK), which was further identified as a novel binding partner of SRGN for the formation of a SRGN/MDK/CD44 complex. In addition, SRGN interacted with MDK and matrix metalloproteinase 2 in ESCC via its GAG chains, which were mainly decorated with chondroitin sulfate comprising of ∆di-4S and ∆di-6S CS. Clinically, high expression of serum SRGN in serum of patients with ESCC was an independent prognostic marker for poor survival.

**Conclusions**: This study provides the first evidence that elevated serum SRGN has prognostic significance in patients with ESCC, and sheds light on the molecular mechanism by which elevated circulating SRGN in cancer patients might promote cancer progression.

## Introduction

Esophageal cancer is the sixth highest ranking cancer disease in terms of mortality rate 1. One reason for its high mortality rate is that the symptoms are often non-specific at the early stages, so that diagnosis is often delayed until advanced stages when the disease has metastasized. The invasion of cancer cells into the surrounding tissue is a crucial step in metastasis. This process usually involves the interaction between cancer cells and components of the tumor microenvironment. Proteoglycans are important components of the extracellular matrix and have a crucial role in tumor progression 2. A proteoglycan molecule consists of a core protein and glycosaminoglycan (GAG) chains, which afford the possibilities to interact with a broad spectrum of proteins such as growth factors and cytokines. These binding partners allow proteoglycans to exert diverse functions in a context-specific manner 3.

Gene profiling of a highly invasive esophageal squamous cell carcinoma (ESCC) subline previously established in our laboratory (named KYSE410 I-3 cells) showed that serglycin (*SRGN*) was the top upregulated gene 4. SRGN is a proteoglycan that consists of an 18-kDa core protein and several GAG chains. Although initially characterized as an intracellular proteoglycan in hematopoietic cells, SRGN is secreted by a variety of cell types 5,6 and is involved in the packaging of proteins and guiding their binding molecules during secretion under physiological and pathological conditions 7-9. Although immunohistochemical studies showed increased SRGN expression in aggressive breast carcinoma 10,11, nasopharyngeal carcinoma 12, and other types of cancer 13, little is known about the diagnostic or prognostic significance of circulating SRGN in patients with cancer. The significance of SRGN in ESCC has not been documented. This study aims to explore the functions and mechanisms of SRGN in the progression of ESCC.

## Materials and Methods

### Cell lines, inhibitors and recombinant human midkine

Human ESCC cell lines KYSE30, KYSE140, KYSE150, KYSE410 14 (DSMZ, Braunschweig, Germany), and T.Tn 15 were used in this study. The establishment of highly invasive ESCC sublines was conducted using Corning® BioCoat^TM^ Matrigel® Invasion Chamber (Corning, NY, USA) as previously described 4. Cell lines were tested for Mycoplasma and were validated by short tandem repeat profiling. A small-molecule compound, iMDK (Merck, Kenilworth, NJ, USA), was used to inhibit endogenous midkine expression. The MEK inhibitor trametinib (SelleckChem, Houston, TX, USA), which is in phase 4 clinical trials, was used to inhibit MAPK/ERK pathway. Recombinant human MDK protein (rhMDK) was purchased from PeproTech, Rehovot, Israel.

### Gene overexpression and silencing

Expression constructs coding for wild-type *SRGN* (named SRGN), truncated *SRGN* lacking a segment of C-terminus containing the GAG attachment region (designated ∆GAG), and *SRGN* with eight native serine residues in the GAG attachment region mutated to alanine (designated mGAG) were generated. Stable expression cell lines were established through lentiviral infection. F-SRGN and F-∆GAG constructs with a FLAG tag inserted between the exon 1 (the signal peptide sequence) and exon 2 of wild-type *SRGN* and *∆GAG,* respectively*,* were used in co-immunoprecipitation (co-IP) experiments. The pLenti CMV Puro DEST vector (#17452, Addgene, Watertown, MA, USA) was used as control (CON). Coding regions of *MDK*, *MMP2*, and *MMP9* were cloned into c-SFB (containing S protein-FLAG-Streptavidin binding peptide at C-terminal on vector backbone) for co-IP. Gene silencing was achieved by using shRNAs for stable knockdown and siRNAs for transient knockdown, with empty vector pLKO.1 (shCON) and scrambled siRNA sequence (siCON) serving as respective controls. Two shRNA sequences against *SRGN* including shSRGN #1 (5'-CCGGGCAGAGCTAGTGGATGTGTTTCTCGAGAAACACATCCACTAGCTCTGCTTTTT-3'), shSRGN #3 (5'-CCGGCCAGGACTTGAATCGTATCTTCTCGAGAAGATACGATTCAAGTCCTGGTTTTT-3'); and one against *MDK* shMDK #5 (5'-CCGGTGTCTGCTCGTTAGCTTTAATCTCGAGATTAAAGCTAACGAGCAGACATTTTTG-3') were purchased from Sigma-Aldrich, St. Louis, MO, USA, and used to generate stable knockdown cell lines. For transient transfection, four siRNAs targeting *SRGN* (siSRGN #2, SI04273696; siSRGN #3, SI04285995; siSRGN #4, SI04287003; siSRGN #5, SI05482057), three targeting *MDK* (siMDK #1, SI00037107; siMDK #2, SI04435725; siMDK #3, SI02663059) and two targeting CD44 (siCD44 #8, SI03037419; siCD44 #10, SI03098123) from Qiagen (Hilden, Germany) were transfected by using Lipofectamine® RNAiMAX Transfection Reagent (Thermo Scientific™, Waltham, MA, USA) according to the manufacturer's manual.

### Cell viability assay

Resazurin reduction assay was used to assess cell viability. In brief, cells were incubated in 0.02% (w/v) resazurin sodium salt (Sigma-Aldrich) for 2 h at 37 °C. The absorbance was detected at 600 nm on a multilabel plate reader (VICTOR3, PerkinElmer 1420, Waltham, MA, USA).

### Cell invasion and migration assays

ESCC cells were resuspended in serum-free medium and seeded onto the Transwell chambers coated with Matrigel® Matrix (Corning) for invasion assay, and without Matrigel for migration assay, as described previously 16. Medium containing 10% fetal bovine serum (FBS) was added to the lower chamber as a chemoattractant. After 24 h, the cells in the upper chambers were removed with cotton swabs and the membranes were stained with 0.1% (w/v) crystal violet. Five fields (100×) of each insert were randomly captured and the area of stained cells were calculated by using ImageJ software 17.

### Apoptosis assay

Cells were cultured in serum-free medium containing staurosporine (BBI Life Sciences, Shanghai, China) to induce apoptosis. Cells were collected after 16 h and stained using Apoptosis Kit with Annexin V FITC (fluorescein isothiocyanate) and propidium iodide (Thermo Scientific™). The fluorescence signal was measured by flow cytometer Canto II analyzer (BD Biosciences, San Jose, CA, USA) and data were analyzed with FlowJo software (BD Biosciences).

### Collection of conditioned media

Culture medium was changed to serum-free medium when the cells reached over 90% confluence. After incubation for 48 h, the supernatant was collected and centrifuged to remove the cell debris. Conditioned medium (CM) was stored at -80 °C for further analysis. For western blotting, CM was concentrated about 20-fold using Amicon® Ultra - 4 mL Centrifugal Filters Ultracel® - 3K (Millipore, Billerica, MA, USA).

### RNA extraction, cDNA synthesis and real-time polymerase chain reaction

Total RNA was extracted using TRIzol (Thermo Scientific™). The quality and quantity of total RNA were examined by NanoDrop 1000 Spectrophotometer (Thermo Scientific™). Reverse transcription (RT) of total RNA was conducted by using High-Capacity cDNA Reverse Transcription Kit (Thermo Scientific™) for real-time PCR (RT-PCR) and by SuperScript™ III First-Strand Synthesis System (Thermo Scientific™) for cloning. Real-time PCR was performed using SsoAdvanced™ Universal SYBR® Green Supermix (Bio-Rad, Hercules, CA, USA) on CFX96 Real-time PCR system (Bio-Rad). The abundance of mRNA was determined using the ΔΔCT method (where CT is threshold cycle) with glyceraldehyde 3-phosphate dehydrogenase (GAPDH) as internal control. Primers used are listed in **Table S1***.*

### Cell fractionation

Cell fractionation was conducted using the Minute^TM^ Cytoplasmic and Nuclear Extraction Kit (Invent, Plymouth, MN, USA) according to the manufacturer's instructions. GAPDH and lamin A/C served as markers for purity of cytosolic and nuclear fractions, respectively.

### Cycloheximide chase analysis

Cells were incubated with 0.1 mg/mL cycloheximide (CHX, Sigma-Aldrich) in culture medium for up to 6 h. The protein lysates were collected for western blotting analysis.

### *In situ* proximity ligation assay (PLA)

*In situ* protein-protein interactions were detected using the Duolink PLA fluorescence kit (Sigma-Aldrich) according to the manufacturer's instructions. Primary antibodies included mouse anti-SRGN (#sc-393521, Santa Cruz Biotechnology, Dallas, TX, USA), goat anti-MDK (#AF-258-PB, R&D Systems, Inc., Minneapolis, MN, USA), rabbit anti-MMP2 (#ab92536, Abcam, Cambridge, United Kingdom) and rabbit anti-MMP9 (#ab76003, Abcam). Briefly, SRGN and a potential interacting protein were detected with a pair of primary antibodies raised in different species, followed by oligonucleotide-labeled secondary antibodies (PLUS and MINUS PLA probes). Connector oligonucleotides were then applied to hybridize and join the probes that were in close proximity (< 40 nm apart) into a closed circular DNA template for rolling circle amplification in the presence of fluorescent-labeled oligonucleotides to form a distinct fluorescent spot 18. Images were captured using a Carl Zeiss LSM 780 confocal microscope system (Zeiss, Jena, Germany).

### Western blotting and co-immunoprecipitation

Cells were lysed in RIPA Lysis and Extraction Buffer (Thermo Scientific™) containing cOmplete™, Mini, EDTA-free Protease Inhibitor Cocktail (Roche, Basel, Switzerland) and PhosSTOP™ phosphatase inhibitor cocktail (Roche). The protein assay and details of immunoblotting were described previously 19. Immunoreactive signals were detected using Clarity™ Western ECL Substrate (Bio-Rad). Actin and GAPDH were used as loading controls. The primary antibodies used in western blotting included: mouse anti-SRGN (#sc-393521, Santa Cruz Biotechnology), rabbit anti-SRGN (#HPA000759, Sigma-Aldrich), rabbit anti-phospho-MEK1/2 (#9121, Cell Signaling, Danvers, MA, USA), rabbit anti-MEK1/2 (#9122, Cell Signaling), rabbit anti-phospho-ERK1/2 (#4370, Cell Signaling), rabbit anti-ERK1/2 (#4695, Cell Signaling), rabbit anti-phospho-p90RSK T573 (#9346, Cell Signaling), rabbit anti-RSK (#9355, Cell Signaling), mouse anti-CD44 (#3570, Cell Signaling), rabbit anti-p-c-Myc S62 (#ab51156, Abcam), rabbit anti-c-Myc (#5605, Cell Signaling), rabbit anti-MMP2 (#ab92536, Abcam), rabbit anti-MMP9 (#10375-2-AP, Proteintech Group, Inc., Rosemont, IL, USA), rabbit anti-MDK (#500-P171, PeproTech), mouse anti-lamin A/C (#4777, Cell Signaling), rabbit anti-GAPDH (#10494-1-AP, Proteintech Group, Inc.), and mouse anti-actin (#sc-47778, Santa Cruz Biotechnology). For co-IP, cells were lysed in IP lysis buffer (50 mM Tris, 150 mM NaCl, 0.5% NP-40, 5 mM EDTA) containing protease and phosphatase inhibitors. The protein lysate was then incubated with anti-FLAG M2 affinity gel beads (#F1804, Sigma-Aldrich) overnight. Unbound proteins were washed away with IP lysis buffer and the immunoprecipitated proteins were denatured with 2× Laemmli sample buffer.

### Immunohistochemistry

Tissue sections were deparaffinized in toluene and rehydrated in graded ethanol. Heat-induced epitope retrieval was done by incubating the sections in boiling citrate buffer. Endogenous peroxidase was blocked with 3% hydrogen peroxide (Merck) for 1 h. Normal goat serum (10%) was used to block non-specific antibody binding before incubation with rabbit anti-SRGN 5 (a gift from Professor Achilleas D Theocharis, University of Patras, Patras, Greece) or mouse anti-Ki-67 (#M7240, Dako, Carpinteria, CA, USA). The sections were then incubated with EnVision+ System secondary antibody conjugated with the horseradish peroxidase-labelled polymer (#K4002, Dako) and developed using 3,3'-diaminobenzidine substrate Kit (Abcam). Nuclei were counterstained with hematoxylin. The slides were scanned on the Nanozoomer (Hamamatsu Photonics, Hamamatsu, Japan). The staining for SRGN was classified into high (strong staining intensity in > 50% cells) and low (weak staining, and less than 50% and mostly 0 to 10% cells stained). The Ki-67 index was calculated by counting about a thousand cells and determining the percentage of cells that were Ki-67 positive.

### Immunofluorescence staining

Cells were seeded on sterilized coverslips and fixed in 4% paraformaldehyde. After permeabilization in 0.1% Triton X-100 and blocking of non-specific binding with 5% bovine serum albumin, the slides were incubated in a mixture of two primary antibodies (one of which was mouse anti-SRGN) overnight, followed by a mixture of corresponding secondary antibodies in the dark. The other primary antibodies used for co-immunostaining were goat anti-MDK (#AF-258-PB, R&D Systems, Inc., Minneapolis, MN, USA), rabbit anti-MMP2, and rabbit anti-MMP9. The slides were mounted in mounting medium containing 4′,6-diamidino-2-phenylindole (DAPI). Images were captured using a Carl Zeiss LSM 780 confocal microscopy (Zeiss).

### Chemokine and phospho-kinase arrays

Proteome Profiler Human Chemokine Array Kit (R&D Systems) was used to compare 31 different chemokines in the CM of KYSE410-CON, KYSE410-SRGN, and KYSE410-∆GAG cells according to the manufacturer's instructions. Human Phospho-Kinase Array Kit (R&D Systems) was used to compare 45 different kinases in cell lysates of KYSE30-CON and KYSE30-MDK. Immunoreactive signals were detected using Clarity™ Western ECL Substrate (Bio-Rad) and exposed to Amersham Hyperfilm ECL (GE Healthcare, Chicago, IL, USA).

### Isolation and analysis of proteoglycans from conditioned medium

Proteoglycans were isolated from the CM by precipitation with cationic detergent cetylpyridinium chloride, and the precipitates were dissolved in isopropanol. The polyanionic macromolecules were precipitated by adding sodium acetate-saturated ethanol and washed in ethanol. The extract was dissolved in ammonium acetate and then treated with chondroitinase AC (ChAC, Sigma-Aldrich), chondroitinase B (ChB, Sigma-Aldrich), chondroitinase ABC (ChABC, Sigma-Aldrich), heparinase I (NEB, Ipswich, MA, UK), heparinase II (NEB), or heparinase III (NEB) for further analysis.

### Fluorophore-assisted carbohydrate electrophoresis (FACE) analysis

The FACE analysis in this study was based on the principle described previously 20. In brief, proteoglycan preparations were digested with ChAC, ChB, ChABC to produce disaccharide products. The products were tagged by incubation with 500 nmoles aminoacridone (Sigma-Aldrich) and 50,000 nmoles sodium cyanoborohydride overnight in the dark. Glycerol (30%) was added to the reaction mixture for further analysis or for storage at -80 °C in the dark. The tagged disaccharide samples were separated by polyacrylamide gel without sodium dodecyl sulfate, and images were captured by Gel Doc XR + gel documentation system (Bio-Rad).

### Reverse phase protein array (RPPA) analysis

The cell lysates of KYSE30-SRGN, KYSE150-SRGN, KYSE410-SRGN, T.Tn-SRGN, and their corresponding controls were subjected to RPPA analysis. Sample spots were probed with antibodies and visualized by 3,3'-diaminobenzidine colorimetric reaction to produce stained slides 21. All relative protein level data points were normalized for protein loading and transformed to linear values. Heatmap was generated using Cluster 3.0 22 and Java TreeView 23. The top 15% upregulated proteins in *SRGN*-overexpressing cell lines were subjected to Gene Ontology (GO) analysis and PANTHER pathway analysis 24.

### Serum from patients and tissue microarray (TMA)

The serum samples used in this study were collected, with approval from the institutional committee for ethical review of research involving human subjects (IRB number: UW19-643), from 100 patients with ESCC upon admission to the Queen Mary Hospital, Hong Kong, from 2003 until 2017. A TMA block containing 40 cases of esophageal carcinoma with matched lymph node metastasis (ES810b) was bought from US Biomax, Derwood, MD, USA. Two cases with inadequate tumor tissue or non-squamous tumor were excluded in the analysis. The remaining 38 cases were all ESCC.

### Enzyme-linked immunosorbent assay (ELISA)

Human serglycin ELISA kit (#SEC869Hu, Cloud-Clone Corp., Katy, TX, USA) and human midkine ELISA Kit (#ab193761, Abcam) were used to determine the concentration of human serglycin and midkine in serum samples of patients with ESCC.

### Tumor xenograft and metastasis experiments

Two ESCC cell lines (i.e. KYSE150 and KYSE410) were used in tumor xenograft experiments. About 5 × 10^5^ cancer cells with *SRGN* knockdown (shSRGN #3) or shCON as control were suspended in equal volumes of PBS and Matrigel and injected subcutaneously into the right flank of 6-week-old BALB/c female nude mice (n = 6/group) to establish the tumor xenografts. During the experiment, the tumor volume was measured every 3 days and calculated with the equation V = (length × width^2^) / 2. Tumor wet weight was assessed at the end point (Day 15 for KYSE150, and Day 21 for KYSE410). At the end of the experiments, the tumors were dissected and stained with hematoxylin and eosin (H&E) and immunostained for analysis of Ki-67 proliferation index. For experimental metastasis model, about 3.5 - 5 × 10^5^ luciferase-expressing KYSE150-Luc cells were intravenously injected into nude mice via the tail vein. Metastasis was assessed by bioluminescent imaging (Xenogen IVIS 100 *in vivo* imaging system, PerkinElmer, Hopkinton, MA, USA). One experiment consisted of a *SRGN*-overexpressing (SRGN) group and a vector control (CON) group. In a second experiment, the experimental groups included *SRGN-*overexpressing (SRGN + shCON), SRGN with *MDK*-knockdown (SRGN + shMDK #5), *SRGN-*knockdown (CON + shSRGN #3) and control (CON + shCON). All the experiments performed in this study were approved by the Committee on the Use of Live Animals in Teaching and Research of the University of Hong Kong.

### Analysis of gene expression and survival data from cancer patient datasets

The gene expression data from The Cancer Genome Atlas (TCGA) and Genotype-Tissue Expression (GTEx) were analyzed using Gene Expression Profiling Interactive Analysis (GEPIA) to compare gene expression in tumor with that in normal samples 25. The survival curves and gene correlation results were in part based on data generated by the TCGA Research Network: https://www.cancer.gov/tcga. Microarray gene expression of cohorts of ESCC patients were downloaded from the Gene Expression Omnibus (GEO) database (accession numbers GSE23400, GSE75241, GSE47404) 26. For GSE23400 and GSE75241, gene expression of *SRGN* in tumor was compared with in the matched tumor-adjacent normal tissues. For GSE47404, gene expression of *SRGN* in primary tumor was compared for patients with and without lymph node metastasis.

### Statistical analysis

All data of *in vitro* experiments are presented as the mean ± standard deviation from at least three independent experiments. Paired or unpaired Student's *t*-test was performed to compare differences between two samples or groups when data had a normal distribution. One-way analysis of variance (ANOVA) was used to compare differences between multiple groups. Pearson's correlation analysis was used to determine the correlation between serum SRGN and serum MDK concentrations. Fisher's Exact test was used to determine the association between clinicopathological characteristics and expression of SRGN or MDK. Overall survival curves were displayed as Kaplan-Meier curves, and survival rates of patients with high versus low expression of SRGN/MDK (segregated using median expression level as cut-off value) were compared using Log-rank test. The Cox proportional hazards model was used to identify whether the factors were independent prognostic factors. Results were presented as hazard ratio (HR) with 95% confidence interval (CI). All statistical operations were performed using Statistical Product and Service Solutions (SPSS) 25.0 Statistics (IBM SPSS, Inc., Chicago, IL, USA). A *P* value of < 0.05 was considered significant (*, *P* < 0.05; **, *P* < 0.01; ***, *P* < 0.001).

## Results

### High *SRGN* expression in ESCC is associated with cancer invasion, poor patients' survival rate and metastasis

**Figure 1:**
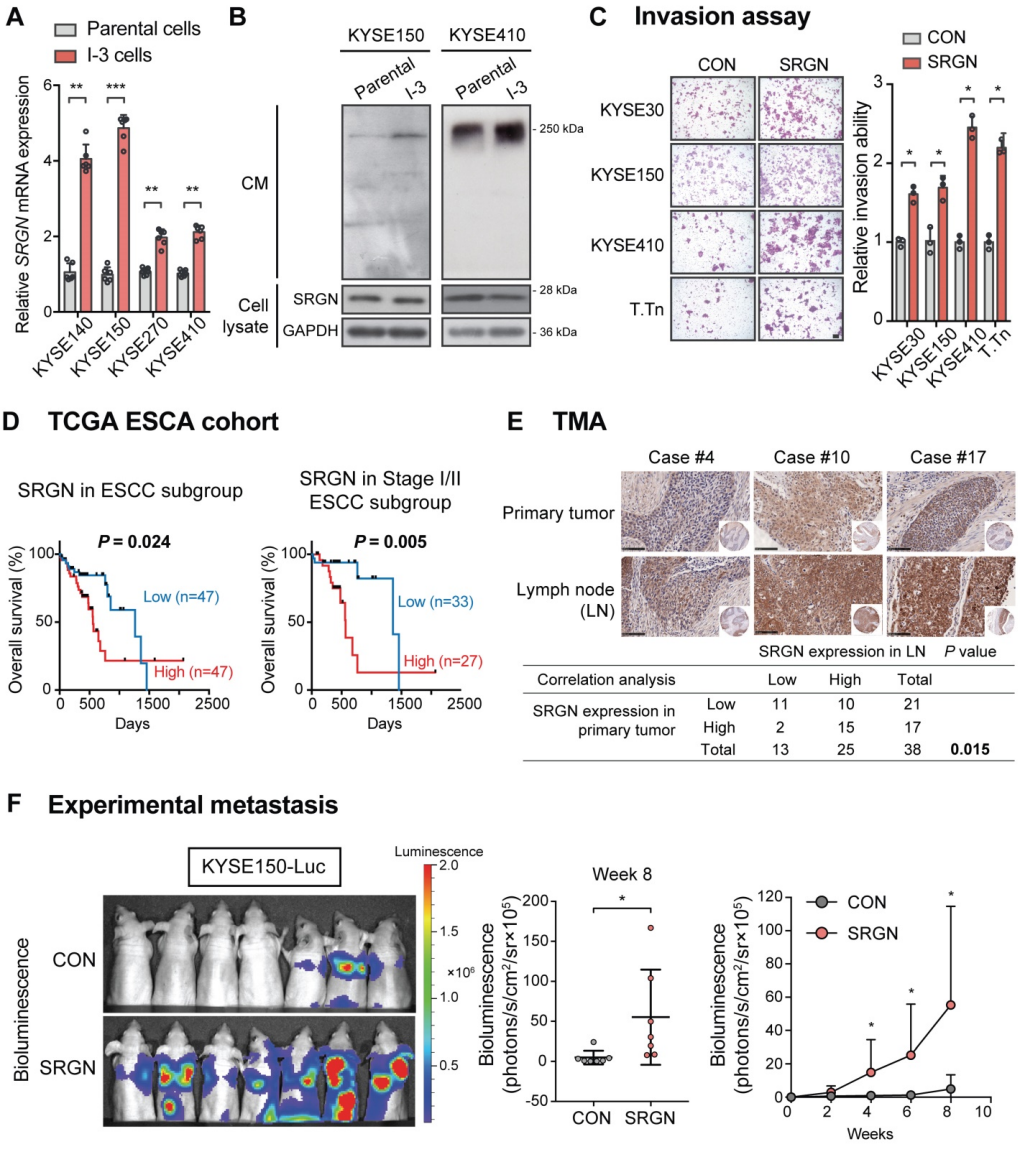
** SRGN expression in human ESCC samples and the effects of SRGN overexpression on ESCC cells *in vitro* and *in vivo*.** (A) The gene expression of *SRGN* in parental and I-3 cells was evaluated by RT-PCR. (B) Western blot analysis of SRGN in CM and cell lysates of I-3 cells compared with corresponding parental cells. (C) Invasive ability of four *SRGN*-overexpressing ESCC cell lines were compared with corresponding vector controls using transwell invasion assay. Scale bar, 100 µm. (D) Kaplan-Meier survival curves of 94 patients with ESCC from TCGA (left panel) and the subgroup of patients with stage I/II ESCC (right panel) segregated according to high and low expression of *SRGN*. (E) Representative images of immunohistchemical staining for SRGN in primary ESCC tumor tissue and matched lymph node (LN) metastasis tissue (upper panel; scale bar, 100 µm). Lower panel shows Fisher's exact test correlation analysis of SRGN expression in the primary tumor and LN metastasis. (F) Experimental metastasis assay in nude mice showing the effect of *SRGN* overexpression on colonization of KYSE150-Luc cells to the lungs (n = 7/group).

Highly invasive sublines (designated as I-3 cells) were newly established from three ESCC cell lines after three rounds of *in vitro* invasion through Matrigel-coated chambers (**Figure S1A**). RT-PCR confirmed that *SRGN* mRNA expression was significantly upregulated in all sublines compared with the corresponding parental cells (**Figure 1A**), which was consistent with what we reported previously for KYSE410-I-3 cells 4. Western blotting results showed that the expression of SRGN protein was increased in the CM of the I-3 cells although there was no obvious change in the cell lysates (**Figure 1B**). Then, four ESCC cell lines with stable overexpression of wild-type *SRGN* were generated. The expression was validated by RT-PCR and western blotting (**Figure S1B-C**). In the western blotting (**Figure S1C**), a major band of SRGN at ~28 kDa was apparent, sometimes as doublets, in the cell lysates of all *SRGN*-overexpressing cell lines. Smeared bands (typical of proteoglycans) ranging from 17 kDa to 250 kDa were observed in the CM. These *SRGN*-overexpressing cells showed increased invasive ability compared with corresponding control cells expressing empty vector (**Figure 1C**).

Kaplan-Meier survival analysis of the TCGA esophageal carcinoma (ESCA) cohort showed that high expression of *SRGN* in tumor was associated with poorer survival (**Figure S2A**). The same phenomenon was found in the TCGA pan-cancer cohort (**Figure S2B**), in which ESCA and many other cancer types were found to have higher *SRGN* expression compared with non-neoplastic tissue (**Figure S2C**). In the ESCC subgroup of the TCGA ESCA, an inverse correlation between high *SRGN* expression and survival time was apparent in patients with Stage I or II ESCC (**Figure 1D)**, suggesting that *SRGN* might have a role in the early stage of development of ESCC. Analysis of GEO datasets revealed that *SRGN* was not only overexpressed in ESCC compared with adjacent non-neoplastic tissue, but its expression was significantly higher in ESCC with lymph node metastasis than those without (**Figure S2D**). Furthermore, immunohistochemical analysis of SRGN expression in a TMA showed a positive correlation between SRGN expression in lymph node metastases and that in primary ESCC (**Figure 1E**). Notably, high expression of SRGN was found in 65.8% (25/38) of lymph node metastasis samples, versus 44.7% (17/38) of primary tumors, which suggests that *SRGN* might be involved in metastasis. In an experimental metastasis model, bioluminescence imaging (**Figure 1F**) showed that intravenously injected KYSE150-Luc cells with *SRGN* overexpression had a superior ability to metastasize to the lungs of nude mice, relative to the control cells.

### Knockdown of *SRGN* can suppress tumorigenic hallmarks in ESCC

**Figure 2:**
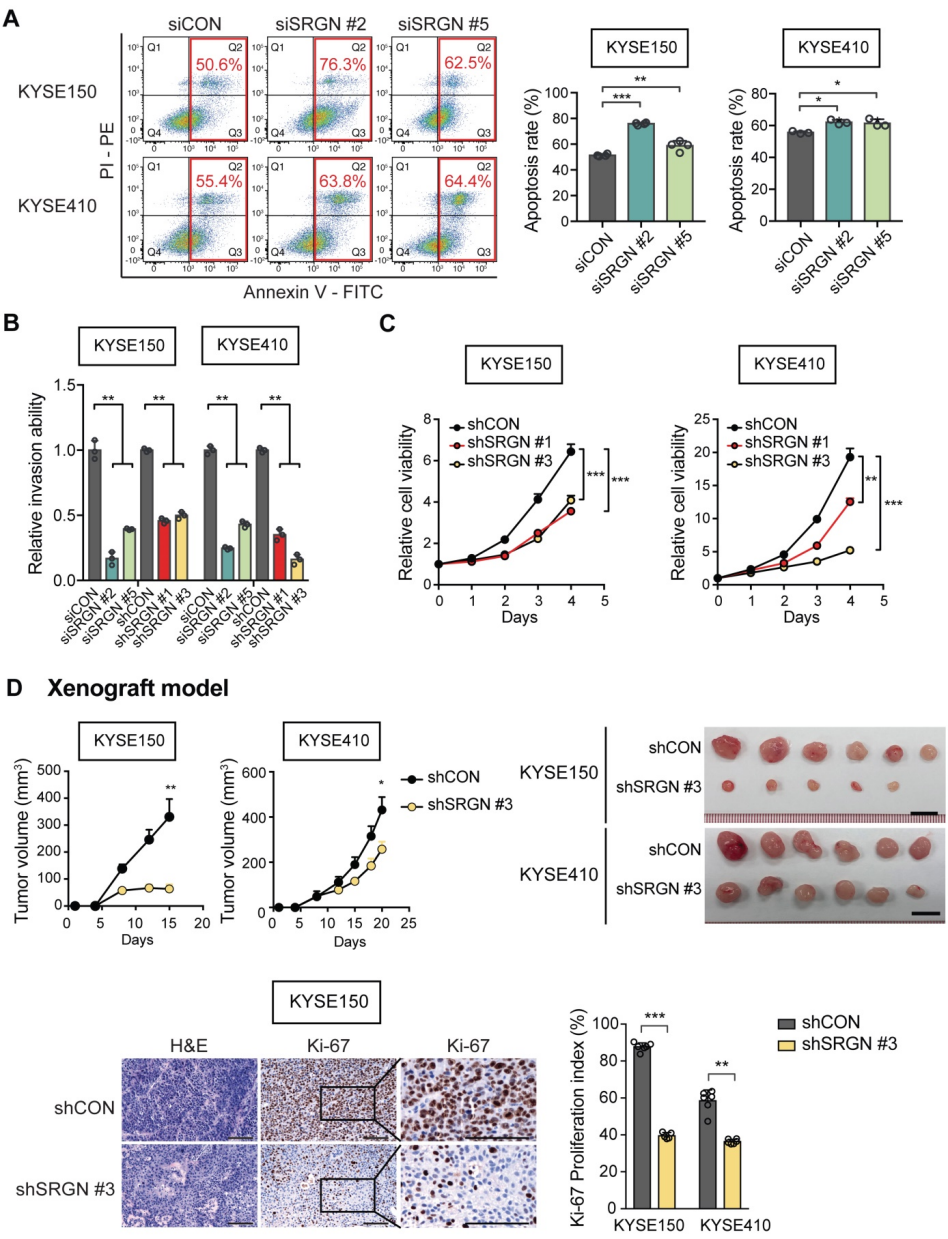
***SRGN*-knockdown suppresses malignant potential of ESCC cells *in vitro* and *in vivo*.** (A) Flow cytometry was used to compare apoptosis in *SRGN*-knockdown cells and control cells. (B) Inhibitory effect of *SRGN*-knockdown by siRNA and shRNA on invasion of ESCC cells. (C) Reduced viability of ESCC cells with *SRGN*-knockdown, compared with vector controls. (D) Effects of *SRGN*-knockdown on growth of ESCC tumor xenografts. Upper panels show tumor volume at different time points (n = 6/group), and the excised tumors at endpoint of experiment (15 days and 20 days after subcutanous injection of KYSE150 and KYSE410 cells, respectively). Lower panels show representative sections of tumor xenografts with H&E staining and Ki-67-immunostaining, and Ki-67 proliferation index of subcutaneous tumor xenografts at endpoint of experiment. Scale bar, 200 µm.

In loss-of-function experiments with *SRGN*-knockdown (**Figure S3A-B**), KYSE150 and KYSE410 cells showed increased staurosporine-induced apoptosis (**Figure 2A**), reduced invasive ability (**Figure 2B and Figure S3C**) and viability (**Figure 2C**). Furthermore, knockdown of *SRGN* greatly decreased the invasiveness of KYSE410-I-3 cells to levels lower than that of the parental cells (**Figure S3D**). In the *in vivo* tumorigenicity assay, stable knockdown of *SRGN* significantly decreased tumor growth rate and proliferative activity (**Figure 2D** and**Figure S3E**). Taken together, these data show that SRGN plays an important role in promoting and sustaining the carcinogenesis of ESCC.

### SRGN promotes cancer cell invasion in an autocrine and GAG-dependent manner

**Figure 3:**
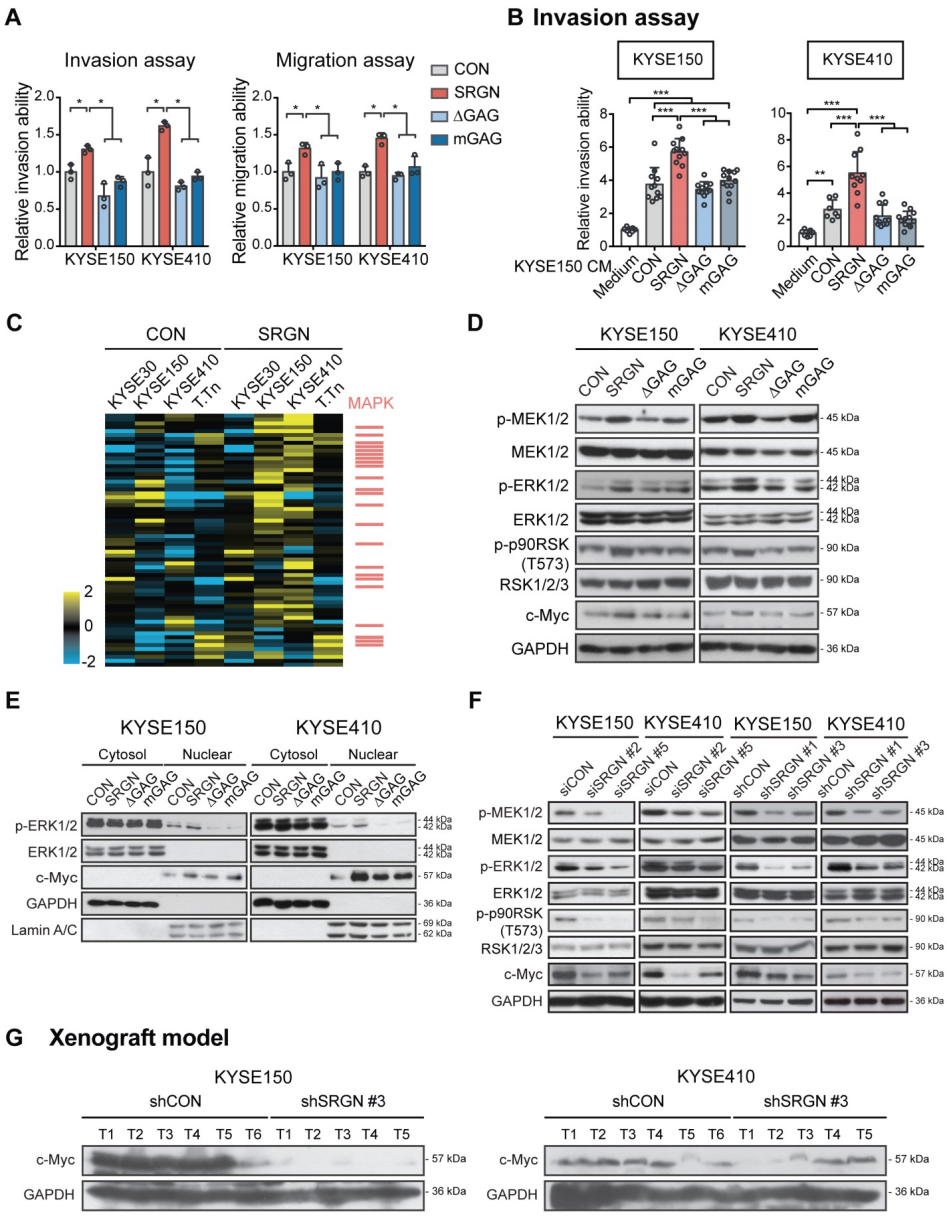
**Significance of GAG attachment domain of SRGN in its promotion of ESCC cell invasion, ERK activation and c-Myc upregulation.** (A) Effects of forced expression of intact *SRGN*, *SRGN* with truncated GAG (*∆GAG*), and mutated GAG (*mGAG*) on invasion (left panel) and migration (right panel) of ESCC cells. (B) Transwell invasion assay showing the effects of the CM collected from KYSE150-*SRGN* cells as chemoattractant on invasion of KYSE150 and KYSE410 cells, compared with CM from *∆GAG*, *mGAG*, and CON cells. (C) Heatmap of reverse phase protein array (RPPA) analysis performed on cell lysates of four ESCC cell lines with *SRGN* overexpression compared with their corresponding control cells. The red bars on the right side indicate the proteins associated with the MAPK pathway. (D) Western blotting analysis of MEK/ERK pathway cascade and c-Myc in ESCC cells expressing *SRGN, ∆GAG, mGAG*, and CON. (E) Western blotting of subcellular fractions (cytosol and nuclear) of ESCC cells with *SRGN, ∆GAG, mGAG* overexpression. (F) Effect of *SRGN-*knockdown on MEK/ERK pathway cascade and c-Myc expression in ESCC cells. (G) Comparison of c-Myc expression in tumor xenografts established from *SRGN*-knockdown cells and vector control cells.

To study whether the pro-invasive function of SRGN was GAG-dependent, SRGN expression constructs with truncated (∆*GAG*) and mutated GAG (*mGAG*) attachment region were designed (**Figure S4A**). They were successfully expressed in the ESCC cell lines (**Figure S4B-C**). In the western blots (**Figure S4C**), glycosylated SRGN (appearing as a smear) was found in the CM of the cells expressing wild-type *SRGN*, while SRGN core protein was detected at ~28 kDa in the cell lysates and CM of both wild-type *SRGN-* and *mGAG*-expressing cells. No band was detected for ∆GAG because the antibody used was designed to recognize the C-terminal of SRGN core protein proximal to the GAG attachment region. *In vitro* assays showed that perturbation of the GAG attachment domain abolished the increased invasiveness and migratory potential of *SRGN*-overexpressing ESCC cells (**Figure 3A** and**Figure S4D**). Cell viability was not affected (**Figure S4E**). The CM from the *CON-*, *SRGN*-, *∆GAG-* and* mGAG*-expressing cell lines were then used as a chemoattractant in an invasion assay. The results showed that the CM of KYSE150-SRGN cells could significantly induce the invasion of KYSE410 and KYSE150 cells in comparison with that of *CON-*, *∆GAG-* and *mGAG-*expressing cells (**Figure 3B** and**Figure S4F**). Together, these data indicate that *SRGN* can exert autocrine pro-invasive effects on ESCC cells, and that the GAG-binding domain of SRGN is crucial in this function.

### SRGN stabilizes c-Myc by autocrine activation of ERK pathway

To decipher the signaling pathway(s) affected by *SRGN* upregulation, four ESCC cell lines were subjected to RPPA analysis, which measured the expression levels of 436 key cancer-related proteins. The top 15% upregulated proteins in *SRGN*-overexpressing cells (**Table S2**) were subjected to PANTHER Pathway and Gene Ontology (GO) analyses. Both analyses indicated that these proteins were significantly associated with the regulation of the MAPK cascade (**Figure S5A-B**) rather than the AKT pathway (**Figure S5C**). **Figure 3C** shows a heatmap of the RPPA results with MAPK pathway-associated factors indicated as red bars on the right. Western blotting showed increased phospho-ERK1/2 (p-ERK1/2) and p-p90RSK (T573), an immediate downstream effector of ERK1/2, in the total cell lysates of KYSE150-SRGN and KYSE410-SRGN cells, but not in the ∆GAG and mGAG cells (**Figure 3D**). Further analysis indicated that p-ERK1/2 was increased in the nuclear fraction of *SRGN*-expressing cells (**Figure 3E**). Stable expression of wild-type *SRGN* also resulted in elevation of c-Myc expression in the total cell lysate and nuclear fraction (**Figure 3D-E**). Conversely, *SRGN-*knockdown suppressed ERK pathway activation (**Figure 3F**). There was also a very marked reduction in the expression of c-Myc in these cells (**Figure 3F**) and in the tumor xenografts derived from them (**Figure 3G**).

**Figure 4:**
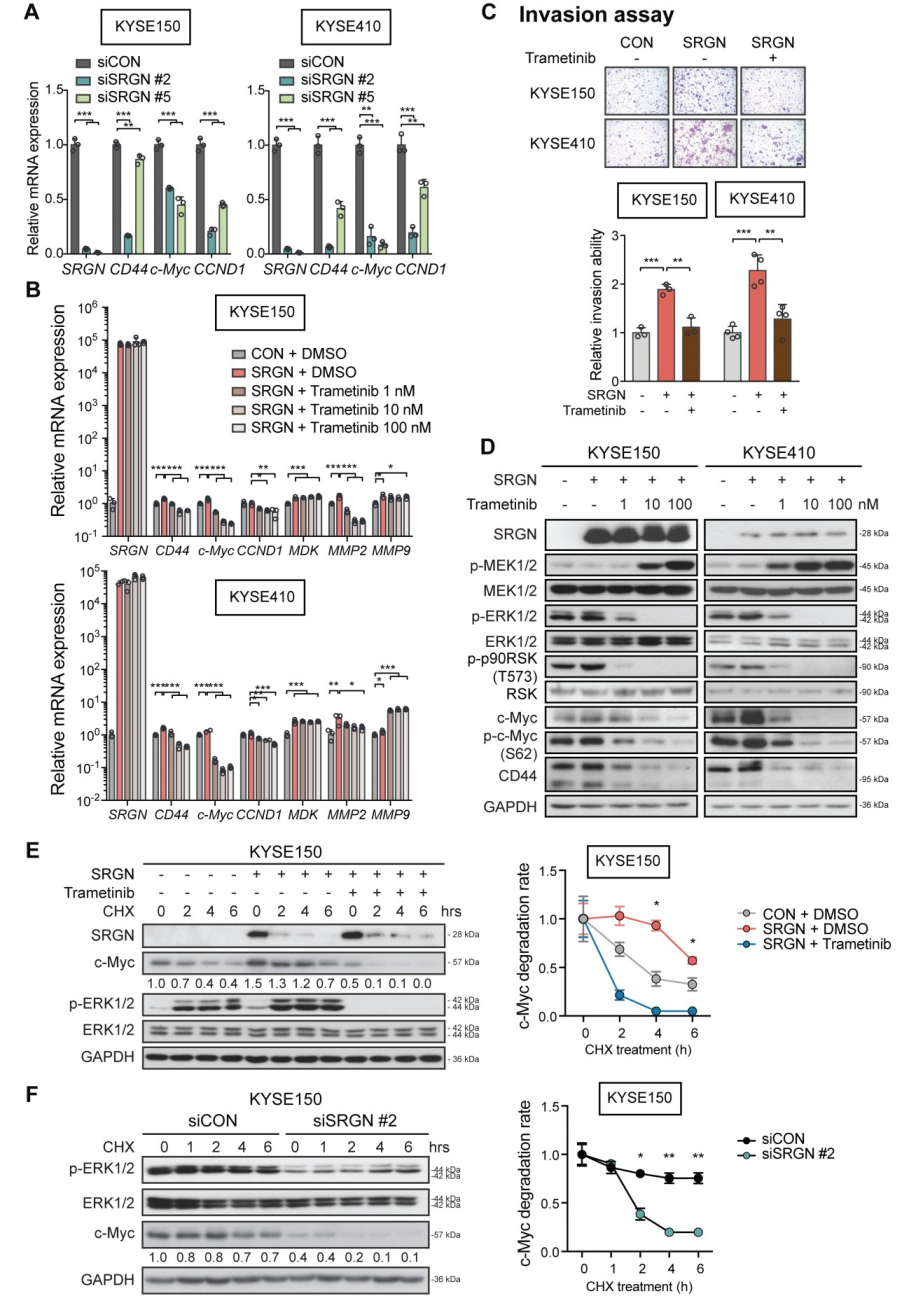
** Significance of ERK pathway in SRGN-induced cell invasion and c-Myc stabilization.** (A) Effects of *SRGN*-knockdown on mRNA expression of *SRGN*, *CD44*, *c-Myc*, *CCND1* in ESCC cells. (B) *SRGN*-overexpressing ESCC cells were treated with trametinib (at indicated concentrations) for 24 h, and the *SRGN* mRNA expression level evaluated by RT-PCR. Cells treated with DMSO served as control. (C) *SRGN*-overexpressing ESCC cells were treated with 100 nM trametinib for 48 h, and then evaluated by invasion assay (upper panel, scale bar, 100 µm). The quantification is shown in the lower panel. (D) *SRGN*-overexpressing cells were treated with increasing concentrations of trametinib as indicated for 72 h. The expressions of members of the MEK/ERK pathway cascade and c-Myc were evaluated by western blotting. Untreated vector controls were included for comparison. (E) KYSE150-SRGN cells with or without trametinib (100 nM) treatment were incubated in 100 µg/mL cycloheximide (CHX) for indicated duration before western blotting analysis (left panel). The numbers below the c-Myc blots are the band intensities of c-Myc that were normalized against GAPDH and then expressed relative to that at 0 h time point. The relative c-Myc degradation rate is presented in the graph (right panel). (F) KYSE150 cells with *SRGN-*knockdown were subjected to CHX chase assay and the band intensities of c-Myc were quantified. CHX chase assay results of KYSE410 cells are shown in Figure S6A-B.

Furthermore, the mRNA expression of ERK downstream factors *CD44*, *c-Myc* and *CCND1* was significantly reduced in *SRGN-*knockdown cells (**Figure 4A**). Treatment with trametinib, which is a selective allosteric inhibitor of the MEK1 and MEK2 (MEK1/2) activity, had no effect on *SRGN* mRNA expression (**Figure 4B**) but counteracted *SRGN*-induced cell invasion (**Figure 4C**), as well as caused dose-dependent p-ERK1/2 inhibition and c-Myc downregulation (**Figure 4D**). The results indicate that SRGN regulates c-Myc through the ERK pathway. Western blotting also showed an increase in MEK1/2 phosphorylation (p-MEK1/2) after trametinib treatment (**Figure 4D**), which could be due to relief of negative feedback phosphorylation.

In the CHX chase assay, the stability of c-Myc protein was increased in *SRGN*-overexpressing cells but greatly reduced in *SRGN*-knockdown cells (**Figure 4E-F** and**Figure S6A-B**). In addition, treatment with trametinib abolished the stabilizing effect of SRGN on c-Myc, indicating that SRGN enhanced the stability of c-Myc by activating ERK. Because the CM from *∆GAG-* and *mGAG-*expressing cells, or heat-inactivated CM from *SRGN*-expressing cells had no effect on p-ERK1/2 (**Figure S6C**), activation of p-ERK1/2 by SRGN and the subsequent stabilization of c-Myc were likely to be autocrine and GAG-dependent.

### SRGN-induced MDK mediates the pro-invasive and ERK/c-Myc-upregulating effects of SRGN

**Figure 5:**
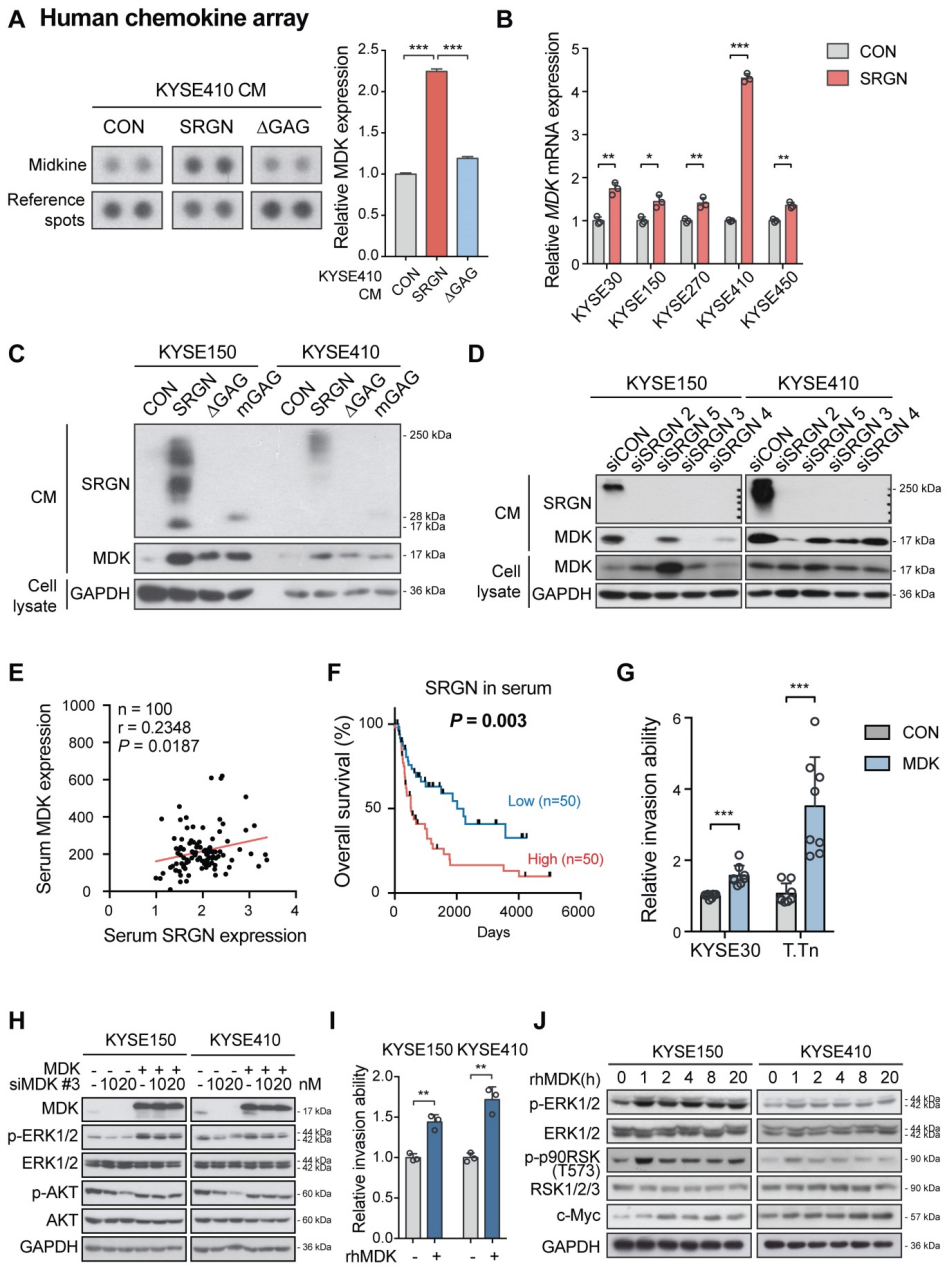
** SRGN-induced MDK mediates the pro-invasive and ERK/c-Myc-upregulating effects of SRGN.** (A) Chemokine profiling of CM from KYSE410 cells expressing control vector (CON), *SRGN* and *∆GAG*, respectively. The intensity of the MDK spots was quantified and presented in the right panel. (B) RT-PCR results showing the effect of *SRGN* overexpression on mRNA expression of *MDK* in ESCC cells. (C) The expression of MDK in the CM of ESCC cells with *SRGN, ∆GAG, mGAG* overexpression was evaluated by western blotting. GAPDH in the cell lysates was used as loading control. (D) The expression of MDK in CM and cell lysates of ESCC cells with* SRGN* knockdown was compared with that of vector control cells. (E) Correlation between serum SRGN and serum MDK in 100 patients with ESCC. (F) Kaplan-Meier curves comparing the survival outcome of patients with high versus low serum SRGN expression. (G) Effect of *MDK* overexpression on invasion of KYSE30 and T.Tn cells. (H) Effects of *MDK* overexpression on p-ERK1/2 and p-AKT expression in KYSE150 and KYSE410 cells. Cells were first transfected with siMDK #3, which targeted the 3'UTR of *MDK*, to knockdown *MDK* and then MDK was re-expressed by overexpression of *MDK.* (I) Effect of rhMDK (500 ng/mL) on invasion of KYSE150 and KYSE410 cells. (J) KYSE150 and KYSE410 cells were treated with 500 ng/mL rhMDK for indicated duration before analysis of MEK/ERK pathway cascade by western blots.

A multiplex chemokine antibody array was used to identify differentially expressed chemokines secreted by *SRGN*-overexpressing ESCC cells. The results showed that there was a significant increase in MDK expression in the CM of KYSE410-SRGN cells (**Figure 5A** and**Figure S7A**). SRGN-induced upregulation and secretion of MDK were further confirmed in multiple ESCC cell lines using RT-PCR (**Figure 5B**) and western blotting (**Figure 5C** and**Figure S7B**). Knockdown of *SRGN* decreased MDK in the CM (**Figure 5D**). Notably, although *∆GAG*- and *mGAG*-expressing ESCC cells also showed elevated MDK in the cell lysates compared with control cells, the MDK expression in their CM was low compared with that of *SRGN*-expressing cells (**Figure 5C** and**Figure S7B)**. However, knockdown of *SRGN* did not consistently decrease mRNA expression of *MDK* in KYSE150 and KYSE410 (**Figure S7C**). Next, we analyzed SRGN and MDK concentrations in serum samples of 100 patients with ESCC using ELISA for correlation analysis and found that they were positively correlated (**Figure 5E**). In addition, serum SRGN expression was found to be significantly correlated with pT-stage in patients with ESCC (**Table S3)**. Kaplan-Meier survival curves showed that patients with higher serum SRGN had significantly poorer overall survival rates (**Figure 5F**). Multivariate analysis showed that SRGN level in admission serum was an independent prognostic factor (**Table 1**).

**Figure 6:**
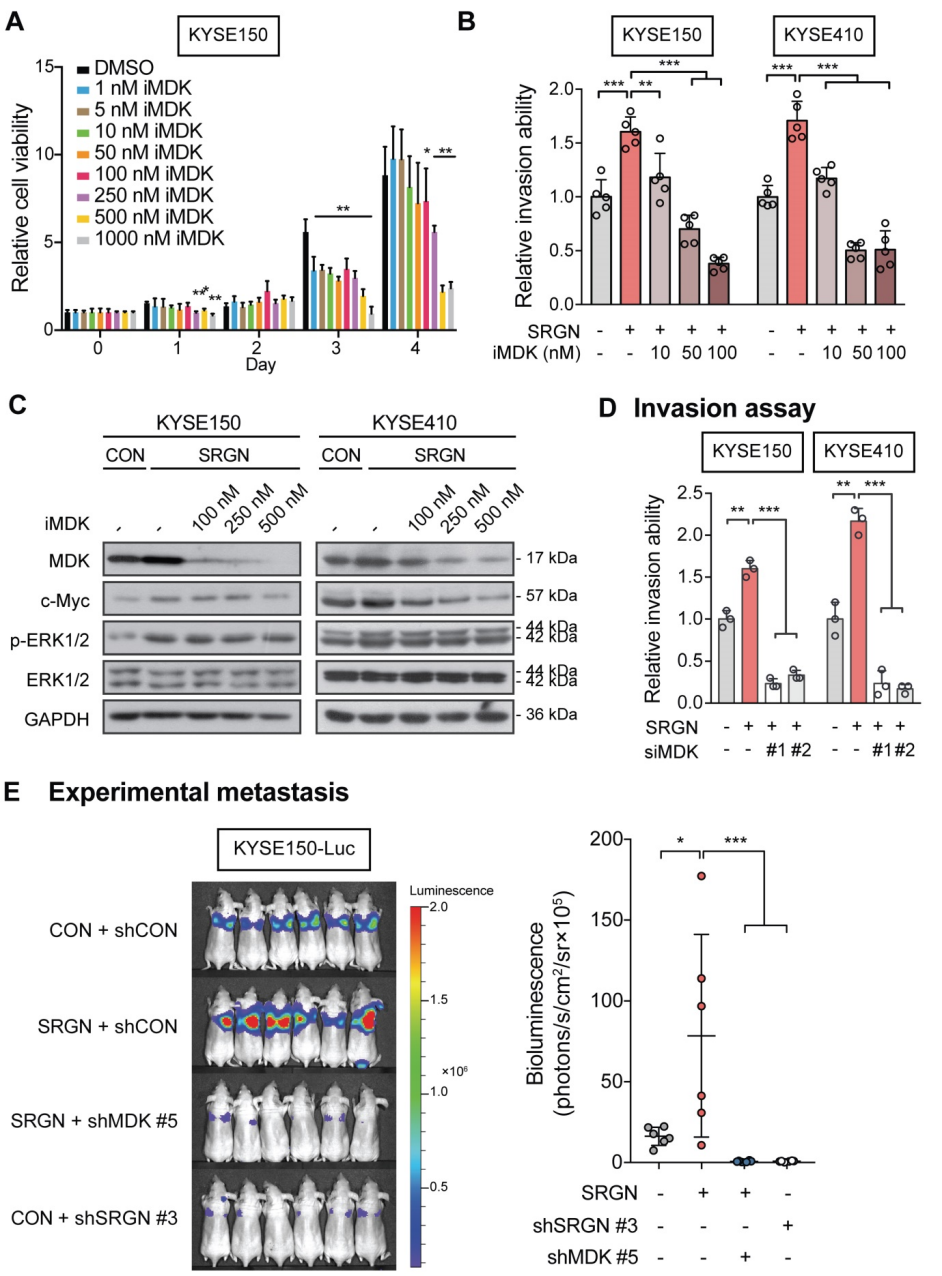
** Effects of MDK suppression on SRGN-induced malignancy of ESCC cells *in vitro* and *in vivo*.** (A) Cell viability of KYSE150 cells treated with MDK inhibitor (iMDK) at indicated concentrations. (B) The effect of iMDK (10, 50 and 100 nM) on invasion of *SRGN*-overexpressing cells was evaluated using transwell invasion assay. (C) Effect of iMDK treatment (at indicated concentrations for 72 h) on c-Myc and ERK phosphorylation in *SRGN*-overexpressing cells. (D) Effect of *SRGN* overexpression together with *MDK*-knockdown by siRNA on invasion of ESCC cells. (E) Bioluminescence imaging and quantification of lung metastasis in nude mice (n = 6 /group) 4 weeks after intravenous injection of KYSE150-Luc cells with manipulated *SRGN* and *MDK* expressions. The *SRGN* overexpression (SRGN + shCON), *SRGN* overexpression with *MDK*-knockdown (SRGN + shMDK #5), and *SRGN*-knockdown (CON + shSRGN #3) groups were compared with the control group (CON + shCON).

The function of MDK in cancer cell invasion *in vitro* was then examined. Increased invasive ability **(Figure 5G** and**Figure S7D**) and p-ERK1/2 expression (**Figure 5H** and**Figure S7E-F**) were observed in ESCC cells with forced expression of *MDK*. Knockdown of *MDK* by using siMDK #3, which targeted the 3'UTR of *MDK*, reduced p-ERK1/2 and p-AKT expression (**Figure 5H**). Exogenous overexpression of *MDK* in *MDK*-knockdown cells could restore the expression of p-ERK1/2 and p-AKT, indicating that the reduction of p-ERK1/2 and p-AKT was indeed caused by the loss of MDK (**Figure 5H**). Moreover, treatment with rhMDK had a potent promoting effect on invasion of ESCC cells (**Figure 5I** and**Figure S7G**) and activation of ERK pathway (**Figure 5J**). Western blot analysis showed that p-ERK1/2 and p-p90RSK (T573) expressions in the ESCC cells exposed continuously to rhMDK (up to 20 h) increased rapidly within 1 h of treatment, and that the levels were sustained until the end of the experiment (**Figure 5J**). The c-Myc expression level was upregulated throughout the duration of treatment (**Figure 5J**). This stimulatory effect of rhMDK on ERK pathway could be abolished by *CD44*-knockdown or *SRGN*-knockdown (**Figure S7H**), indicating that the autocrine stimulatory effect of MDK on ERK pathway relies on SRGN and CD44. However, when the MEK/ERK pathway in *SRGN*-overexpressing ESCC cells was suppressed by treatment with trametinib, leading to downregulation of known downstream effectors of MEK/ERK pathway such as *CD44*, *c-Myc,* and *CCND1*, there were no significant changes in the mRNA expression of *MDK* (**Figure 4B**), inferring that MDK is not a direct downstream effector of the MEK/ERK pathway. Treatment with iMDK, a cell-permeable imidazothiazolyl-chromenone compound that selectively inhibits endogenous MDK (**Figure S8A**), decreased the viability of ESCC cells in a dose-dependent manner (**Figure 6A** and **Figure S8B**). It also reversed the effects of SRGN on ESCC cell invasion (**Figure 6B** and**Figure S8C**). Consistent with that observed in non-small cell lung cancer 27 and oral squamous cell carcinoma 28, iMDK reduced p-AKT expression in KYSE150 and KYSE410 (**Figure S8D**). Interestingly, iMDK also decreased protein level of c-Myc in *SRGN*-expressing cells in a dose-dependent manner, even though the p-ERK1/2 expression level was not suppressed (**Figure 6C**). Knockdown of *MDK* in *SRGN*-overexpressing cells not only reduced ESCC cell viability (**Figure S8E**) and invasion *in vitro* (**Figure 6D** and**Figure S8F**), but also suppressed lung metastasis *in vivo* to the same extent as *SRGN*-knockdown (**Figure 6E**). Taken together, these data showed that SRGN upregulates MDK expression in ESCC cells and promotes its secretion in a GAG-dependent manner, and that SRGN-induced MDK contributes to cancer cell invasion.

### MDK binds to chondroitin sulfate GAG chains of SRGN and forms a complex with SRGN and CD44

**Figure 7:**
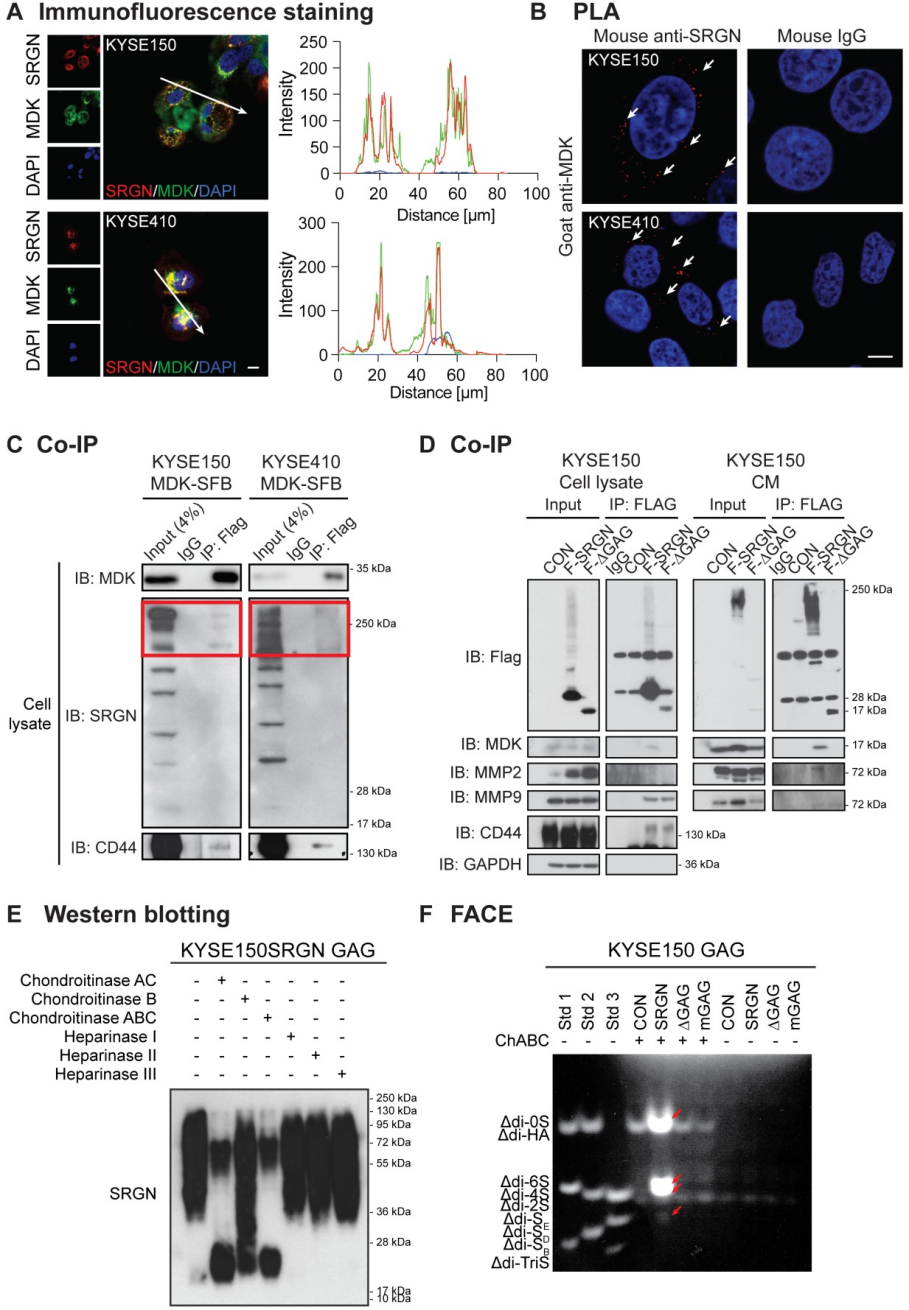
** MDK binds to glycosylated SRGN.** (A) Typical images of ESCC cells immunostained for SRGN (red) and MDK (green), and counterstained with 4',6-diamidino-2-phenylindole (DAPI) are shown in the left panel (scale bar, 10 µm). Line-scan profiles of immunofluorescence signals along the white arrows showed co-localization of SRGN and MDK. (B**)** Proximity ligation assay (PLA) for SRGN and MDK was performed on KYSE150 and KYSE410 cells. After ligation and amplification, the nuclei were counterstained with DAPI. Red spots (indicated by white arrows) represent the binding between SRGN and MDK proteins. Negative control was conducted by replacing SRGN antibody with mouse IgG. Scale bar, 10 µm. (C) Whole cell lysates of MDK-SFB transfected ESCC cells were immunoprecipitated with anti-FLAG M2 beads before immunoblotting for cellular SRGN and CD44. Western blotting showed that FLAG-fusion MDK co-precipitated with SRGN at ~250 kDa (red frames), and with CD44 at over 130 kDa. (D) Cell lysates and CM of FLAG-fusion *SRGN*-overexpressing (F-SRGN) and FLAG-fusion *∆GAG*-expressing (F-∆GAG) cells were incubated with anti-FLAG M2 beads. The core protein of SRGN was predominantly precipitated in cell lysate and glycosylated SRGN was predominantly precipitated in CM. Western blots showed that MDK and MMP2 were bound to glycosylated SRGN, but not to SRGN core protein. MMP9 and CD44 were bound to both glycosylated SRGN and core protein. (E) Proteoglycans isolated from CM of KYSE150-SRGN cells were digested with the indicated enzymes before detection of SRGN using western blotting. (F) Fluorophore-assisted carbohydrate electrophoresis (FACE) analysis of disaccharide products in the CM of KYSE150 cells expressing *CON*, *SRGN*, *ΔGAG* and *mGAG*, with or without predigestion with chondroitinase ABC. Lanes Std1, Std2, and Std3 contained the standard markers. ∆di-0S, ∆di-6S, ∆di-4S and ∆di-S_E_ in the CM of *SRGN*-expressing cells are indicated by red arrows.

MDK is a heparin-binding cytokine capable of binding to chondroitin sulfate (CS) proteoglycan 29. Immunofluorescence staining and PLA showed that SRGN and MDK were co-localized and interacted with each other in the cytoplasm of ESCC cells (**Figure 7A-B**). Co-IP experiments were performed to further confirm the interaction between SRGN and MDK. The results showed that endogenous SRGN in the cell lysates co-precipitated with overexpressed MDK-SFB at ~250 kDa (red frames) which was consistent with the size of glycosylated SRGN (**Figure 7C**). We also conducted a co-IP experiment using the cell lysates and CM of ESCC cells overexpressing FLAG-fusion SRGN (F-SRGN) and FLAG-fusion ∆GAG (F-∆GAG), and found that MDK was co-precipitated with SRGN in *F-SRGN*-overexpressing cells but not *F-∆GAG*-overexpressing cells (**Figure 7D** and**Figure S9A**). These co-IP results therefore showed that MDK was bound to glycosylated SRGN. SRGN is a known ligand of CD44 that binds to it through the GAG chains 30. As shown in **Figure 7D** and**Figure S9A**, CD44 (band most obvious at over 130 kDa) was co-precipitated with both F-SRGN and F-∆GAG. Surprisingly, endogenous CD44 also co-precipitated with MDK-SFB in cell lysates (**Figure 7C**). Based on these findings, we conclude that intracellular and secreted MDK binds to the GAG chains of SRGN, and that SRGN, MDK and CD44 form a complex.

To identify the type(s) of GAG chains associated with SRGN secreted by ESCC cells, GAGs were isolated from the CM of *SRGN*-overexpressing ESCC cells, and then digested using chondroitinases and heparinases which selectively degrade CS/dermatan sulfate (DS) GAGs and heparin/heparin sulfate (HS) chains respectively. SRGN core protein was detected after digestion with ChAC and ChABC, while partial digestion was observed after ChB treatment (**Figure 7E** and**Figure S9B**), indicating that the secreted SRGN was mainly decorated with CS GAG chains and, to a lesser degree, DS. FACE analysis showed that ∆di-4S and ∆di-6S were both increased in the CM of KYSE150-SRGN and KYSE410-SRGN cell lines in comparison with those in ∆GAG, mGAG and CON cells (**Figure 7F** and**Figure S9C**), while ∆di-0S and ∆di-S_E_ were also increased in the CM of KYSE150-SRGN cells (**Figure 7F**). The results suggest that a diversity of CS GAG chains are present in SRGN expressed in ESCC cells.

### SRGN upregulates and binds to matrix metalloproteinases

**Figure 8:**
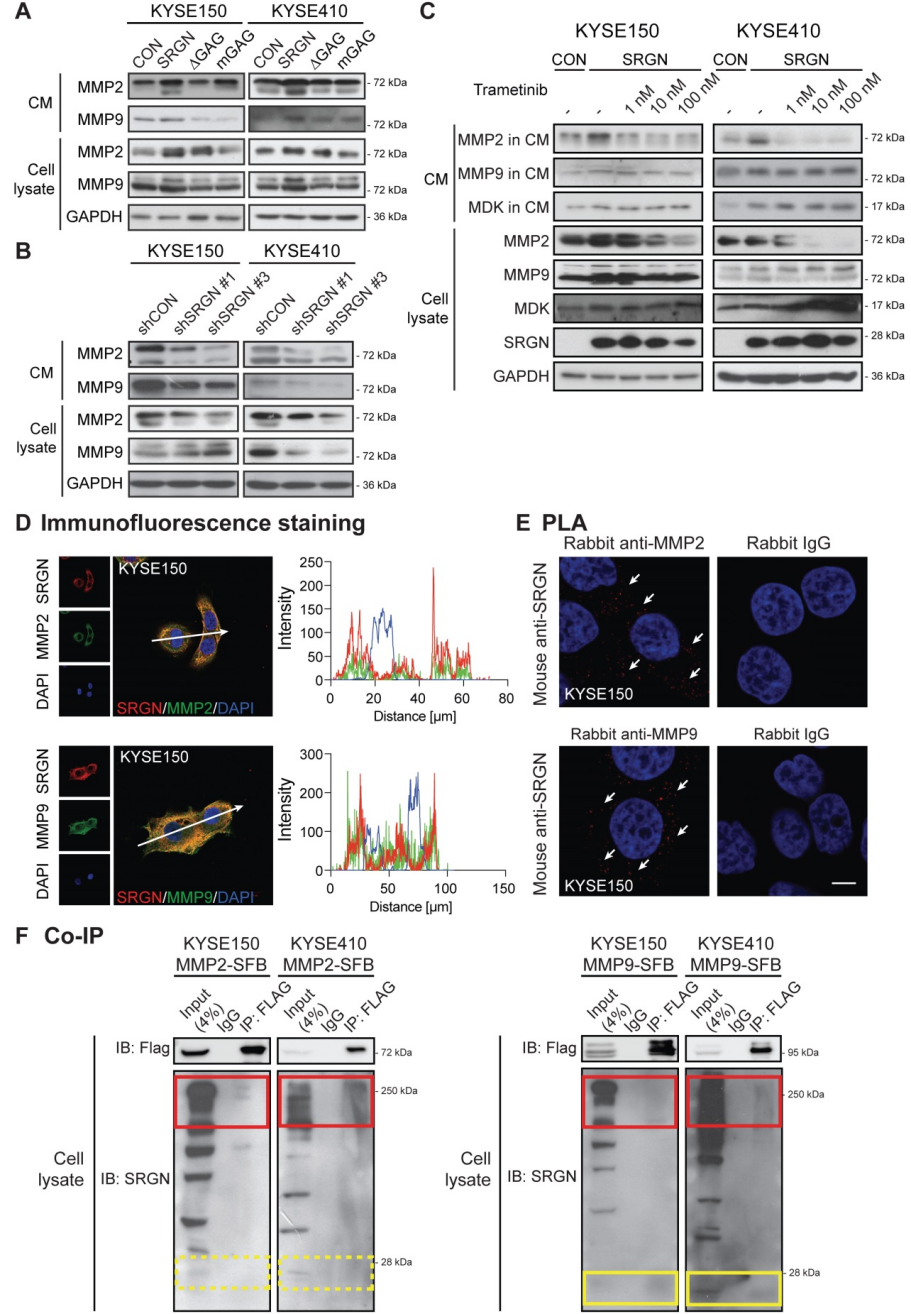
** SRGN upregulates and binds to MMP2 and MMP9.** (A) Effect of *SRGN, ∆GAG, mGAG* overexpression on the expression of MMP2 and MMP9 in cell lysates and CM. (B) Effect of *SRGN-*knockdown on the expression of MMP2 and MMP9 in cell lysates and CM. (C) Western blot analysis of MMP2, MMP9, MDK in cell lysates and CM of *SRGN*-overexpressing cells treated with increasing concentrations of trametinib for 72 h. Untreated vector control cells were included for comparison. (D) Representative images of KYSE150 cells immunostained for SRGN (red) and MMP2 or MMP9 (green) are shown in the left panel. Scale bar, 10 µm. Line-scan profiles of immunofluorescence signals along the white arrows were acquired which showed co-localization between SRGN and the two MMPs (right panel). (E) Representative PLA images (with DAPI counterstain) confirmed the interaction between SRGN and MMP2/MMP9 in KYSE150 cells (white arrows). Negative control was conducted by replacing MDK antibody with rabbit IgG. The images of another cell line KYSE410 are presented in Figure S10B. (F) Whole cell lysates of MMP2-SFB (left panel) and MMP9-SFB (right panel) transfected cells were immunoprecipitated by anti-FLAG M2 beads before immunoblotting for SRGN. Western blotting showed that glycosylated SRGN at ~250 kDa co-precipitated with MMP2 and MMP9 (red frames), whereas the core protein of SRGN at ~28 kDa co-precipitated with MMP9 (yellow frames) but not with MMP2 (yellow frames with dashed lines).

Matrix metalloproteinases (MMPs) are essential components of the extracellular matrix that play important roles in extracellular matrix remodeling during tumor invasion and metastasis. MMP2 and MMP9 are frequently upregulated in many types of cancer including ESCC 31. In the current study, MMP2 and MMP9 were increased in the CM of I-3 cells (**Figure S10A**), which prompted us to determine whether SRGN might be involved in regulating the secretion of these enzymes. Western blotting results confirmed that MMP2 and MMP9 were indeed elevated in the CM of ESCC cells overexpressing wild-type SRGN (**Figure 8A**) and decreased in CM of *SRGN*-knockdown cells (**Figure 8B**). Since these MMPs were not elevated in the CM of *∆GAG*- and *mGAG*-expressing cells, their induction by SRGN might be GAG-dependent. MMPs were reported to be regulated by MAPK pathway 32. Interestingly, unlike MMP2, the increase of MMP9 in cell lysates and CM of *SRGN*-overexpressing cells (**Figure 8C**), and *MMP9* mRNA expression (**Figure 4B**) was not abolished by treatment with trametinib. This suggests that SRGN did not upregulate MMP9 via the MEK/ERK pathway. Immunofluorescence staining and PLA were used to further explore the relationship between SRGN and MMP2/MMP9. As presented in **Figure 8D-E** and**Figure S10B-C**, SRGN was co-localized and interacted with both MMP2 and MMP9 in the cytoplasm of KYSE150 and KYSE410 cells. In addition, co-IP experiments showed that overexpressed MMP2-SFB co-precipitated with glycosylated SRGN in the cell lysates, while MMP9-SFB co-precipitated with core protein of SRGN at ~ 28 kDa as well as with glycosylated SRGN (**Figure 8F**). This difference was further confirmed in **Figure 7D** and**Figure S9A**, which show that MMP9 was co-precipitated with SRGN in cell lysates of both *F-SRGN*- and *F-∆GAG*-overexpressing cells, while MMP2 was mainly co-precipitated with SRGN in CM of *F-SRGN*-overexpressing cells. In summary, these results suggest that, in addition to binding with SRGN in the cytoplasm, MMPs may be secreted together with SRGN.

## Discussion

SRGN is unique among proteoglycans in that it is the only intracellular proteoglycan identified so far 33. We found that SRGN was overexpressed in 44.7% of primary ESCC tumors and in over 65% of lymph node metastasis samples. In addition, high expression of SRGN in serum of ESCC patients was associated with poor prognosis. While intracellular SRGN modulates the packaging, secretion, delivery or transportation of proteins such as growth factors, proteases, cytokines/chemokines 3,34, secreted SRGN can interact with CD44 30, MMP9 35, extracellular matrix components 36,37 and pro-inflammatory factors 38,39. However, the mechanisms of SRGN and its molecular interactions during cancer progression are far from being fully understood. In this study, we have provided the first evidence of binding between glycosylated SRGN and MDK in cancer cells and explored its functional significance.

We found that all our *SRGN*-overexpressing ESCC cell lines showed increased MDK expression in the CM even though the intracellular MDK was not consistently elevated, suggesting that SRGN was involved in the process of MDK secretion. The mRNA expression of *MDK* was not affected in *SRGN*-knockdown cells, indicating that MDK is not a direct downstream effector of SRGN. Since SRGN is not a transcription factor, it is possible that a cascade of molecules including MDK are involved in mediating the pro-invasive function of SRGN. ESCC cells exposed to the CM of *SRGN*-overexpressing ESCC cells or treated with rhMDK showed enhanced invasiveness, which suggests a SRGN-driven autocrine stimulatory mechanism. Thus, it is possible that secreted MDK induced by SRGN is involved in mediating the pro-invasive function of SRGN. Our data further revealed that MDK binds to SRGN both intracellularly and extracellularly after secretion, and that the CS GAG chains on SRGN are critical in their interaction. Moreover, SRGN also activated the expression and proteolytic activity of MMP2 and MMP9 in an autocrine manner, which is in line with previous reports 38,40.

Extracellular SRGN is a well-documented ligand for the transmembrane receptor CD44, and GAG chains also serve as binding elements in this interaction 30. Our co-IP analysis showed binding not only between CD44 and SRGN, but also between MDK and CD44 in ESCC cells. A search of the literature revealed that CD44 was required for SRGN to promote aggressiveness of non-small cell lung cancer 41, and that CD44-positive prostate cancer cells were significantly more susceptible to the inhibitory effect of *MDK*-knockdown on cell viability 42. Considering that both soluble and substratum-bound forms of MDK promote growth, survival and migration of various target cells 43, our data suggest that complexing with CD44 might facilitate the autocrine effect of SRGN-induced MDK.

The requirement of GAGs on SRGN in order for it to exert its tumorigenic properties was first reported by Korpetinou *et al.* 10. The current findings highlight the functional significance of the GAG chains of SRGN, which is in agreement with some previous studies 38,41,44. It is well established that different types of GAG chains have different affinity to bind to bioactive molecules 45. Unlike mast cells in which the predominant types of GAG chains of SRGN are HS and CS, or macrophages in which ∆di-S_E_ CS predominates 7,13,34, the GAG chains isolated from the CM of *SRGN*-overexpressing ESCC cells were mainly decorated with ∆di-4S and ∆di-6S CS. This finding was in agreement with what was reported for SRGN secreted by breast cancer and myeloma plasma cells 5,10. These CS chains might contribute to the formation of MDK/CD44 complex in ESCC cells. It is worth noting that HS-GAG was not evident in SRGN secreted by ESCC cells, similar to what was recently reported for non-small cell lung cancer cells 44. It is possible that, in addition to being overexpressed, cancer cell-secreted SRGN may differ from that expressed in normal cells in the composition of GAG chains, such as loss of HS or relative abundance of specific GAG moieties that favor the recruitment of cancer-promoting binding partners. It had been reported that the CS chains of SRGN have better binding capacity to several proteins of the complement system (especially in collagen-like modules) than heparin and oversulfated CS chains 46.

The MAPK/ERK and PI3K/AKT pathways are frequently activated by multiple molecular mechanisms in ESCC 4,47,48. Our RPPA results showed upregulation of MAPK/ERK cascade but not the PI3K/AKT pathway in *SRGN*-overexpressing cells. Treatment with rhMDK and CM from *SRGN*-overexpressing cells led to ERK activation in ESCC cells. The lack of such responses in cells treated with CM of *∆GAG*- or *mGAG*-expressing ESCC cells suggests that the GAG side chains on SRGN, and/or their binding partners, such as MDK, are crucial in autocrine activation of ERK pathway. This finding further supports that interactions between GAG chains and chemokines are indispensable for chemokine activity 49. Furthermore, our results showed that the stimulatory effect of rhMDK on ERK pathway was CD44-dependent, indicating that CD44 was the crucial receptor in this autocrine stimulation mechanism. We found that overexpression of *MDK* did activate ERK pathway in ESCC cells. However, inhibition of MDK by iMDK did not reduce p-ERK1/2 activity in *SRGN*-overexpressing cells, which suggests that this autocrine effect may not be fully dependent on MDK. This is reasonable because SRGN can bind to many other biologically important molecules through its GAG chains and/or core protein. On the other hand, this unexpected finding could be due to the property of the inhibitor itself since there are contradictory reports in the literature regarding whether iMDK suppresses ERK in cancer 28,50. Nevertheless, since SRGN can activate the MAPK/β-catenin pathway by interacting with CD44 51, the binding of SRGN and MDK to CD44 could promote activation of ERK pathway. Besides, our results showed that SRGN can alter the expression and stability of c-Myc by regulating ERK signaling. Based on our results showing that c-Myc was reduced in cells treated with trametinib and iMDK, we postulate that c-Myc is an important ERK-dependent downstream effector of the SRGN-MDK/ERK axis in ESCC cells. Apart from this regulatory axis, we also found evidence of a GAG- and ERK-independent mechanism involving upregulation of another SRGN-binding partner, i.e. MMP9, which might contribute to SRGN-induced cancer cell invasiveness.

In conclusion, our findings substantiate that SRGN and its binding partners promote ESCC aggressiveness from multiple aspects, including autocrine activation of intracellular signaling pathway and tumor microenvironment remodeling. In view of the significance of the GAG chains of SRGN and its binding partners, therapeutics that target SRGN itself or its binding partners, or strategies that modify the GAG chains of SRGN where most of the protein interactions / binding take place, would be a new direction in esophageal cancer therapy.

## Supplementary Material

Supplementary figures and tables.

## Figures and Tables

**Table 1:** Univariate and multivariate Cox proportional hazards analysis of clinicopathological parameters and serum SRGN/MDK in the prognosis of patients with ESCC.

Parameters	Univariate analysis		Multivariable analysis	
	HR (95% C.I.)	*P* value	HR (95% C.I.)	*P* value
**Age (years)**				
≤ 60	1.000 (Reference)		-	-
> 60	1.760 (0.949-3.264)	0.073		
**Gender**				
Male	1.000 (Reference)			
Female	0.891 (0.479-1.656)	0.715	-	-
**pT-Stage**				
1	1.000 (Reference)			
2	2.758 (0.970-7.843)	0.057	1.775 (0.336-9.379)	0.499
3	2.589 (0.995-6.736)	0.051	1.348 (0.263-6.909)	0.721
4	7.877 (2.772-22.38)	0.000	3.689 (0.708-19.23)	0.121
**pN-Stage**				
0	1.000 (Reference)			
1	1.027 (0.514-2.056)	0.939	0.512 (0.159-1.649)	0.262
2	1.733 (0.866-3.466)	0.120	0.724 (0.178-2.936)	0.651
3	2.563 (1.200-5.469)	0.015	1.224 (0.334-4.484)	0.760
**M-Stage**				
0	1.000 (Reference)			
1	0.623 (0.224-1.732)	0.365	-	-
**p-Stage grouping**				
I	1.000 (Reference)			
II	2.646 (0.778-9.000)	0.119	1.585 (0.214-11.73)	0.652
III	3.729 (1.139-12.21)	0.030	2.881 (0.263-31.57)	0.386
IV	1.804 (0.401-8.128)	0.441	1.003 (0.100-10.05)	0.998
**Serum SRGN^#^**				
Low	1.000 (Reference)			
High	2.232 (1.297-3.843)	0.005	2.254 (1.216-4.179)	0.010
**Serum MDK^#^**				
Low	1.000 (Reference)			
High	1.466 (0.861-2.496)	0.159		

# The median expression level was used as cut-off value for segregation into high vs low expression.

## Abbreviations

∆di-0S: chondroitin disaccharide ∆di-0S
∆di-2S: chondroitin disaccharide ∆di-2S
∆di-4S: chondroitin disaccharide ∆di-4S
∆di-6S: chondroitin disaccharide ∆di-6S
∆di-HA: hyaluronic acid disaccharide ∆di-HA
∆di-S_B_: chondroitin disaccharide ∆di-(2,4)S
∆di-S_D_: chondroitin disaccharide ∆di-(2,6)S
∆di-S_E_: chondroitin disaccharide ∆di-(4,6)S
∆di-TriS: chondroitin disaccharide ∆di-(2,4,6)S
ChABC: chondroitinase ABC
ChAC: chondroitinase AC
ChB: chondroitinase B
CHX: cycloheximide
ELISA: enzyme-linked immunosorbent assay
CM: conditioned medium
Co-IP: co-immunoprecipitation
CS: chondroitin sulfate
DS: dermatan sulfate
ESCC: esophageal squamous cell carcinoma
FACE: fluorophore-assisted carbohydrate electrophoresis
GAG: glycosaminoglycan
GAPDH: glyceraldehyde 3-phosphate dehydrogenase
GEO: Gene Expression Omnibus
GO: Gene Ontology
HS: heparin sulfate
IF: immunofluorescence
MMP2: matrix metallopeptidase 2
MMP9: matrix metallopeptidase 9
PLA: *in situ* proximity ligation assay
RPPA: reverse phase protein array
RT-PCR: real-time polymerase chain reaction
SRGN: serglycin
TCGA: The Cancer Genome Atlas
TMA: tissue microarray
