# Supplementary material for: Significance of serglycin and its binding partners in autocrine promotion of metastasis in esophageal cancer
Source: Theranostics. 2021 Jan 1;11(6):2722–41. doi: 10.7150/thno.49547 (PMC7806492; doi:10.7150/thno.49547)
Supplement: Supplementary file 1 — Supplementary figures and tables. [file thnov11p2722s1.pdf]

Table S1. List of primers for RT-PCR and cloning.

	Genes	Sequence (5' – 3') Forward	Reverse
RT-PCR			
	SRGN167	CCTCAGTTCAAGGTTATCCTACGC	CGTCTTTGGAAAAAGGTCAGTCCT
	SRGN264	TATCCTACGCGGAGAGCCAGGTAC	TTCCGTTAGGAAGCCACTCCCAGATC
	GAPDH	AAGGTCATCCCTGAGCTGAA	TGACAAAGTGGTCGTTGAGG
	CD44	CAGCTCATACCAGCCATCCA	TGACTGGAGTCCATATCCATCCT
	c-Myc	CTAGGGTGGAAGAGCCG	GCTGCTATGGGCAAAGTT
	CCND1	ATGCCAACCTCCTCAACGAC	TCTGTTCCTCGCAGACCTCC
	MDK	GAGTCGCCTCTTAGCGGAT	GCCGCCCTTCTTCACCTTAT
	MMP2	GGACTTAGACCGCTTGGCTT	GTGTTCAGGTATTGCATGTGCT
	MMP9	CGGTTTGGAAACGCAGATGG	TGGGTGTAGAGTCTCTCGCT
Cloning			
	attB Adapter	GGGGACAAGTTTGTACAAAAAAGCAGGCT	GGGGACCACTTTGTACAAGAAAGCTGGGT
	SRGN	AAAAAGCAGGCTTCGAAGGAGATAGAACCATGAT GATGCAGAAGCTACTCAAATGCA	AGAAAGCTGGGTCATGGTGATGGTGATGGTGTCCAC CTCCAGGTGGGAAAATCCTCTTTT
	ΔGAG	AAAAAGCAGGCTTCGAAGGAGATAGAACCATGAT GATGCAGAAGCTACTCAAATGCA	AGAAAGCTGGGTCATGGTGATGGTGATGGTGTCCAC CTCCGTAGTCCTCAGAAAGTGGGA
	MMP2	GCTCTAGAGCATGCAGAAGTTCTTTGGACTGCCCC	CGGAATTCCGTCAGCCTAGCCAGTCG
	MMP9	GCTCTAGAGCATGAGCCTCTGGCAGCC	CGGAATTCCGCTAGTCCTCAGGGCACTG

Table S2. List of differentially expressed proteins in *SRGN*-overexpressing cells as determined using RPPA analysis.

Name	KYSE30-CON	KYSE30-SRGN	KYSE150-CON	KYSE150-SRGN	KYSE410-CON	KYSE410-SRGN	T.Tn-CON	T.Tn-SRGN
53BP1	0.43972728	0.4531788	0.75541918	1.0591975	1.02129586	1.04910811	1.013502071	1.600740871
ATP5A	1.00651753	0.88767393	0.79015652	1.35722448	0.96113092	1.06519921	1.081298082	1.17734608
Aurora-A	0.61031043	0.68418952	0.79516207	1.08628627	1.48601143	1.32944296	0.468908631	1.146097996
Aurora-ABC_pT288_pT232_pT198	0.80980579	0.86099114	1.02324634	1.59455281	1.08411949	1.05948203	0.679172985	1.248535054
Axl	1.30892975	1.51320335	2.07013647	1.88369622	0.43674888	0.76026535	0.221998277	0.459340651
b-Catenin	0.87383209	0.92710203	1.36249881	2.23281058	0.9565451	0.97484456	0.958144182	1.85681794
B7-H3	1.07669682	1.02138837	0.67168909	0.9265175	0.95930994	1.02667827	0.559159013	1.099941714
Bim	0.78383399	0.63079116	0.94642018	1.16656766	0.997634	0.93330093	1.255419171	1.981082005
BiP-GRP78	1.22973573	1.09085091	0.93754709	1.16110972	0.88703279	0.95272313	1.078238447	1.103087285
BMK1-Erk5_pT218_Y220	0.94344122	0.90367766	1.08664478	1.36571445	1.00023639	1.00094922	1.048775505	1.397781759
Calnexin	1.22948816	1.05709656	0.81613942	1.1656514	0.97886224	0.95228044	0.885448126	1.206129064
Caveolin-1	1.00940903	1.06320734	1.01299284	1.46762497	0.98129791	1.17570404	0.856291861	2.034830802
CD171	1.75919423	1.23116783	6.18225678	9.43735507	0.47432631	0.84211037	0.369558965	0.44508838
CD44	0.97896699	0.96675914	1.09800171	1.79731503	0.9841182	1.09825032	0.818111835	0.994156095
CDK1_pT14	0.72876475	1.12574977	0.37754651	0.903206	1.12167314	1.00263946	0.357378669	0.857011878

Claudin-7	0.93081069	1.05797138	0.47183721	0.74220072	1.19590756	1.33397125	0.576791389	0.909471769
Complex-II-Subunit	1.00266533	0.98882074	0.93780047	0.89254723	0.96511702	1.05556858	1.076806399	1.393049469
Connexin-43	0.9579779	0.98718959	6.54011229	8.08575366	0.80569598	0.92240251	1.136315918	1.123930075
Cox-IV	1.22010536	1.08030611	0.80509159	1.50601293	0.90888698	0.94107498	1.08873728	1.487127633
Cox2	1.02691358	1.13602709	0.69566655	0.74467525	0.78910331	0.89961019	2.576327842	2.197590733
Cyclin-B1	0.6766001	1.01948775	0.78862076	1.26753412	1.22231848	1.03121508	0.483127824	1.392615142
Cyclophilin-F	1.28805472	0.93327273	1.19210613	1.75706735	0.90384667	0.87460953	0.64076369	1.085230433
DDR1	1.175113	0.98625912	1.68389935	2.00775631	0.74118506	0.79986268	0.597690154	1.066245981
DNMT1	0.78443191	0.82242282	0.93391863	1.33591675	0.99867751	0.93224587	1.144960974	2.013719539
EGFR	2.89094632	3.03978148	0.86849823	0.99135526	0.38344879	0.42429651	0.683823859	1.088392742
EphA2	0.96771855	0.92209977	0.84418266	1.20932569	1.37152577	1.48306689	0.803501539	0.974684853
FN14	1.00009485	1.12374695	1.08820463	1.18759728	0.99247515	1.1228153	0.657464931	0.580176304
FOXM1	0.75977251	0.89116277	0.86591765	0.90196542	1.30368407	1.14585438	0.656709759	1.519021712
H2AX_pS139	1.05191924	1.02450235	0.82853848	1.00734926	0.92280285	0.99229617	1.201898502	1.058832434
HER2	0.89357207	1.1168379	0.54491228	0.57362045	11.193563	27.0262313	1.052364722	0.986087196
HER2_pY1248	1.01575049	1.27012171	0.68525284	0.71645442	6.14413538	7.1009455	0.84458508	0.726531972
HER3	1.2507068	1.06564333	0.98720165	1.03904835	0.90599158	0.98998193	0.839302336	1.190274084
Heregulin	0.4379453	0.41891885	0.97196114	1.23326577	1.09163141	1.19543029	0.485890915	1.516562837

Hexokinase-II	0.81710294	1.06317621	1.61820011	1.07823285	0.98132541	0.79885569	0.78395326	1.409838706
Histone-H3_pS10	1.13648449	1.20560463	0.87182914	1.33273404	0.89471053	0.89139816	1.020827586	1.329164844
IGFRb	1.06766281	1.01703795	0.49158966	0.68369386	0.92717374	1.0024208	1.272111372	2.468730773
MCT4	3.81899207	4.19409074	0.802336	1.21274615	0.88211137	1.05010207	0.487613916	0.616540441
MMP14	0.47724401	0.39945751	1.32553838	2.12574005	0.90211358	1.02987849	0.998798343	2.285877356
MSH6	1.02543724	1.2260643	1.00742731	0.91216003	0.95304612	0.88796562	1.090408315	1.719903716
MTCO1	1.02762624	1.13530689	0.30740257	1.42248339	0.34799973	0.39948068	2.233368979	1.168745336
Myosin-IIa	1.00571118	0.86266711	1.18423367	1.63767657	1.38385427	1.58522846	1.026053687	0.774986799
Myosin-IIa_pS1943	0.82910029	0.85267668	1.3287924	1.75661443	1.8210096	2.00628132	0.887104371	0.641641774
Myt1	0.89675934	0.99625602	0.79626164	1.06698597	1.04045427	1.06482141	0.905897653	1.709508915
P-Cadherin	1.15724164	1.12566644	1.08570398	1.20491596	0.63333919	0.6964274	0.960826181	1.549866425
p90RSK_pT573	1.03118253	1.18039014	1.15436808	1.35119832	0.9311024	0.8935664	1.05476038	1.335525629
PDGFRB	0.91028773	0.86481324	0.82336192	1.22155162	0.78102408	1.12016205	1.340116004	1.144844576
PERK	1.2836755	1.18514881	0.71703792	1.04474805	0.90206284	0.93421935	0.547848144	0.91943556
PHLPP	0.55188276	0.62747388	1.8807705	1.96601681	1.12828604	0.86515136	0.558268523	0.982450353
PLK1	0.83901568	1.09005845	0.80117942	1.4593517	1.1279741	0.96817064	0.449912259	2.425331733
PRC1_pT481	1.0224169	1.02448048	1.05486196	1.42490453	1.00777781	1.03700451	1.009785894	1.378636159
Rad23A	0.96501489	1.11592352	1.15886011	1.50856976	0.93471933	0.88537985	1.020664596	1.101064955

Rad51	1.13091781	1.36522088	0.45157791	0.58036755	1.39053724	1.33577253	0.748408797	0.84026029
Rb_pS807_S811	0.89239307	1.02772667	1.06453075	1.00794334	1.10684575	1.02385482	0.78128309	1.630390557
RPA32_pS4_S8	0.96155933	0.96292144	1.04750147	1.33959597	1.06163841	1.03856332	0.832003588	1.344140671
SDHA	1.04283331	0.84223888	0.86887498	1.12299235	0.90720079	1.00049901	1.453656989	2.378107666
SHP-2_pY542	0.94345133	1.07659764	1.01630665	0.97863863	1.13166023	1.3430753	1.092429587	0.770209135
SLC1A5	0.89501487	0.94477736	1.07021232	1.67056136	1.00782268	1.03777123	0.561795602	1.154922654
Smac	0.95453326	1.08683725	1.16321389	1.19978653	0.80022206	0.9710483	1.00861397	1.008485716
Src_pY527	0.46643366	0.5270868	0.82011619	1.04445615	1.05488174	1.05396664	0.989687522	0.986013613
TFRC	1.00176851	1.16369454	0.94176879	2.78248753	1.01089084	0.83342558	1.025226824	1.828186706
TRAP1	1.13995018	1.08590664	0.88512924	1.03251767	0.5129105	0.5850125	1.083161163	1.486275422
TUFM	1.17406821	1.0074255	1.04908338	1.42013786	0.75552715	0.82304721	1.155959645	1.25702584
UQCRC2	1.06991279	0.93392312	0.86793402	1.26006985	0.89501541	0.98013114	1.087810646	1.395443737
Vinculin	0.86458775	1.09200475	1.12057195	0.58261781	0.8140225	1.00863149	1.254174628	1.100271837
YB1_pS102	1.03656359	1.06917237	1.0192937	1.38113013	0.97602737	0.99256134	0.973383864	1.364430635

Table S3. Correlation between serum SRGN expression level and clinicopathological parameters in 100 cases of ESCC.

Parameters	Serum SRGN				Serum MDK			
	Low	High	Total	*P* value	Low	High	Total	*P* value
**Number**	50	50	100		50	50	100	
**Age (years)**								
≤ 60	14	13	27		16	11	27	
> 60	36	37	73	1.000	34	39	73	0.368
**Gender**								
Male	41	36	77		39	38	77	
Female	9	14	23	0.342	11	12	23	1.000
**pT-Stage**								
1	16	6	22		14	8	22	
2	9	11	20		9	11	20	
3	19	25	44		21	23	44	
4	6	8	14	**0.042**	6	8	14	0.529
**pN-Stage**								
0	22	20	42		20	22	42	
1	15	13	28		16	12	28	
2	7	12	19		10	9	19	
3	6	5	11	0.626	4	7	11	0.674
**M-Stage**								
0	45	47	92		44	48	92	
1	5	3	8	0.715	6	2	8	0.140
**p-Stage**								
I	10	5	15		10	5	15	
II	13	18	31		14	17	31	
III	22	24	46		20	26	46	
IV	5	3	8	0.722	6	2	8	0.192
**Differentiation (grade)**								
G1	2	2	4		3	1	4	
G2	30	29	59		26	33	59	
G3	18	19	37	0.855	21	16	37	0.286

Supplementary Figures

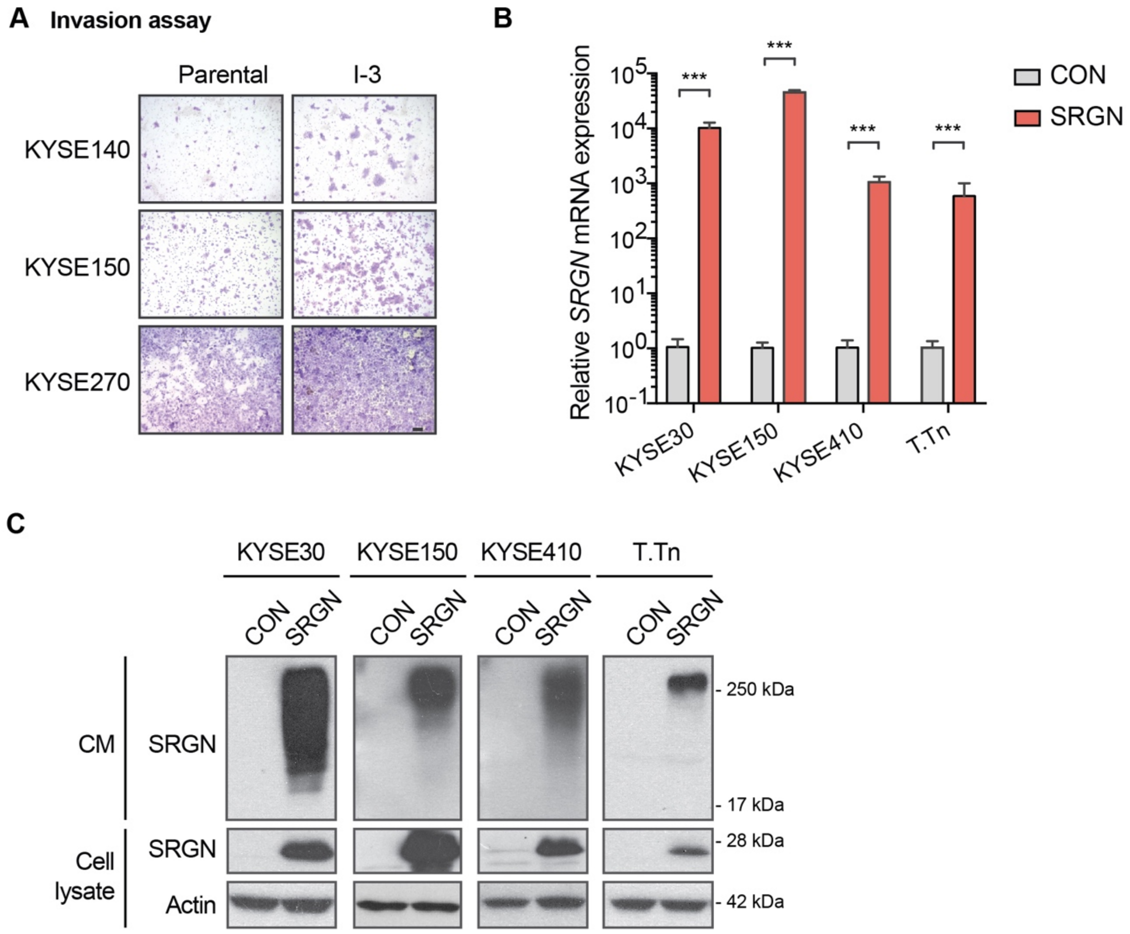

Figure S1. SRGN is upregulated in highly invasive ESCC cells. (A) Representative images of invasion assay comparing three highly invasive ESCC sublines (I-3) with their parental cells. Scale bar, 100 μm. (B-C) Validation of four ESCC cell lines with *SRGN* overexpression by RT-PCR (B) and western blotting (C).

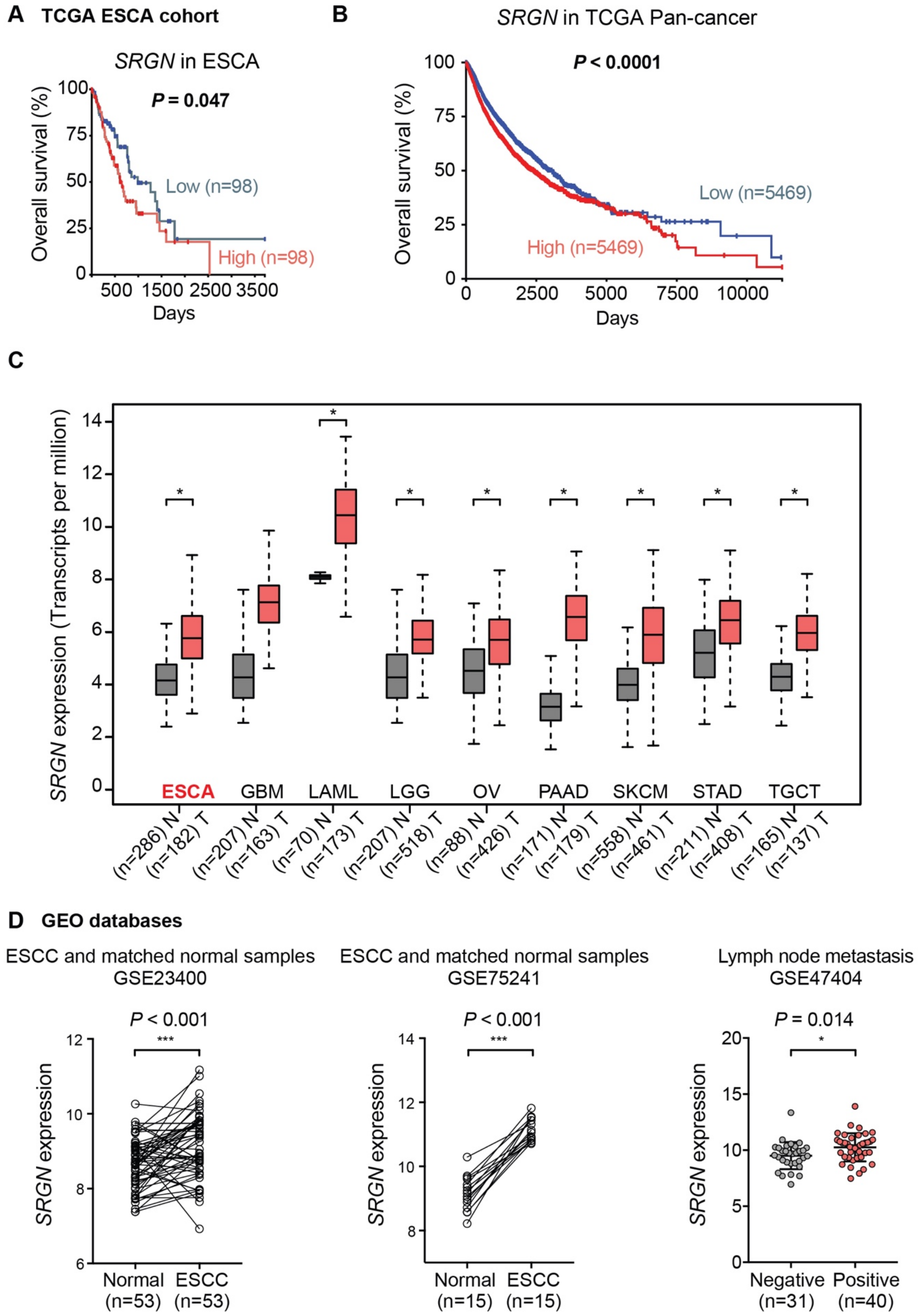
A TCGA ESCA cohort
SRGN in ESCA
P = 0.047
Overall survival (%)
Low (n=98)
High (n=98)
Days
B
SRGN in TCGA Pan-cancer
P < 0.0001
Overall survival (%)
Low (n=5469)
High (n=5469)
Days
C
SRGN expression (Transcripts per million)
ESCA
GBM
LAML
LGG
OV
PAAD
SKCM
STAD
TGCT
(n=286) N
(n=182) T
(n=207) N
(n=163) T
(n=70) N
(n=173) T
(n=207) N
(n=518) T
(n=88) N
(n=426) T
(n=171) N
(n=179) T
(n=558) N
(n=461) T
(n=211) N
(n=408) T
(n=165) N
(n=137) T
D GEO databases
ESCC and matched normal samples
GSE23400
P < 0.001
SRGN expression
Normal (n=53)
ESCC (n=53)
ESCC and matched normal samples
GSE75241
P < 0.001
SRGN expression
Normal (n=15)
ESCC (n=15)
Lymph node metastasis
GSE47404
P = 0.014
SRGN expression
Negative (n=31)
Positive (n=40)

Figure S2. Prognostic significance of SRGN in esophageal cancer and its expression pattern in a wide variety of human cancers including ESCC. (A) Kaplan–Meier plots of survival time in The Cancer Genome Atlas (TCGA) ESCA cohort of patients (n = 196) with high vs low expression of SRGN in the primary tumor (segregated using median expression level as cut-off value). (B) Kaplan–Meier estimates of survival time in TCGA pan-cancer (n = 10,938) grouped according to high vs low *SRGN* expression level. (C) *SRGN* expression in several cancer types (data extracted from TCGA cohorts) compared with normal samples using Gene Expression Profiling Interactive Analysis (GEPIA). ESCA, esophageal carcinoma; GBM, glioblastoma multiforme; LAML, acute myeloid leukemia; LGG, lower grade glioma; OV, ovarian serous cystadenocarcinoma; PAAD, pancreatic adenocarcinoma; SKCM, skin cutaneous melanoma; STAD, stomach adenocarcinoma; TGCT, testicular germ cell tumors. (D) Comparison of *SRGN* expression in human ESCC tissue samples and matched non-neoplastic tissue in GEO datasets GSE23400, GSE75241; and in ESCC with and without lymph node metastasis in GSE47404.

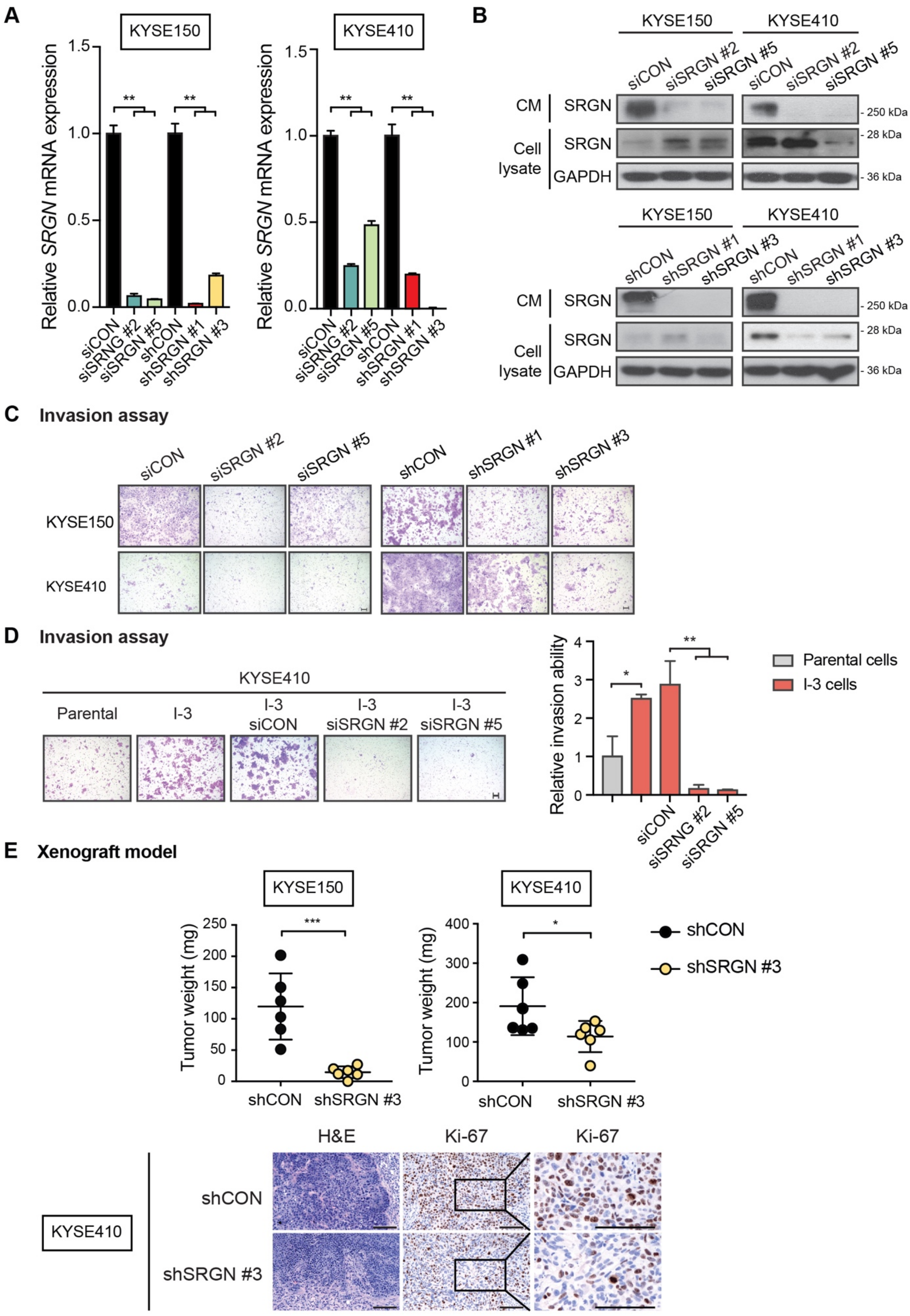
A
KYSE150
Relative SRGN mRNA expression
1.5
1.0
0.5
0.0
**
**
siCON
siSRNG #2
siSRGN #5
shCON
shSRGN #1
shSRGN #3
KYSE410
B
KYSE150
KYSE410
siCON
siSRGN #2
siSRGN #5
CM
SRGN
Cell lysate
GAPDH
- 250 kDa
- 28 kDa
- 36 kDa
shCON
shSRGN #1
shSRGN #3
C
Invasion assay
D
Invasion assay
KYSE410
Parental
I-3
I-3 siCON
I-3 siSRGN #2
I-3 siSRGN #5
Relative invasion ability
4
3
2
1
0
*
Parental cells
I-3 cells
E
Xenograft model
Tumor weight (mg)
250
200
150
100
50
***
400
300
*
shCON
shSRGN #3
H&E
Ki-67

Figure S3. Knockdown of *SRGN* suppresses malignant potential of ESCC cells *in vitro* and *in vivo*. (A) Validation of *SRGN*-knockdown in ESCC cell lines using RT-PCR. (B) Expression of SRGN in cell lysates and CM in *SRGN*-knockdown cells. (C) Representative images of transwell invasion assay showing the effect of *SRGN*-knockdown on invasion in ESCC cells. (D) Representative images (left panel) and quantification (right panel) of the effect of *SRGN*-knockdown on invasion ability of KYSE410 I-3 cells. Scale bars, 100 µm. (E) Effect of *SRGN*-knockdown on tumor xenografts. Upper panels show tumor wet weight at the end of experiment. Lower panel shows representative sections of tumor xenografts with hematoxylin and esoin (H&E) staining and Ki-67-immunostaining. Scale bar, 200 µm.

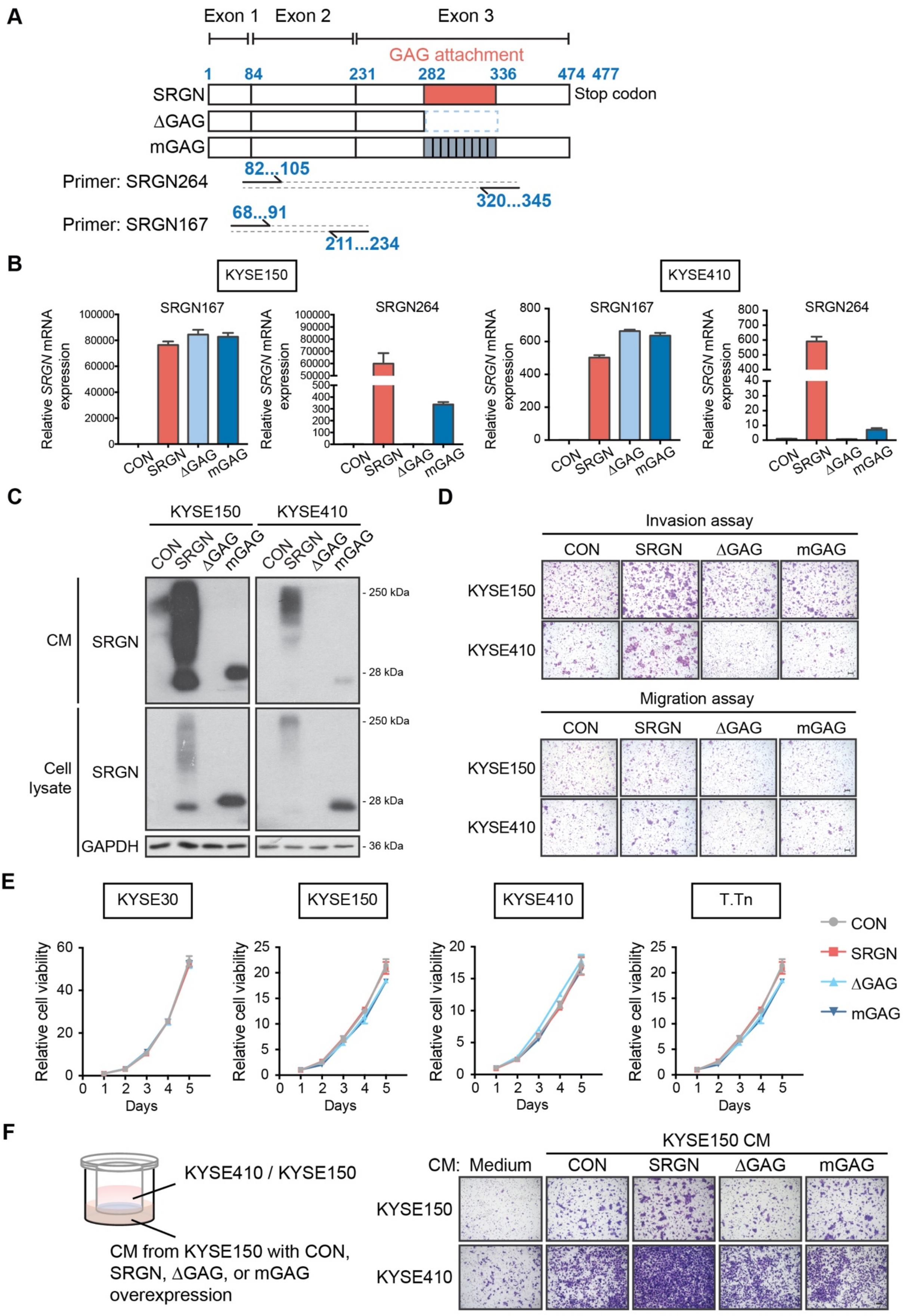
A
Exon 1
Exon 2
Exon 3
GAG attachment
1
84
231
282
336
474
477
SRGN
Stop codon
ΔGAG
mGAG
Primer: SRGN264
82...105
320...345
Primer: SRGN167
68...91
211...234
B
KYSE150
KYSE410
SRGN167
SRGN264
Relative SRGN mRNA expression
CON
SRGN
ΔGAG
mGAG
C
KYSE150
KYSE410
CM
SRGN
Cell lysate
GAPDH
250 kDa
28 kDa
36 kDa
D
Invasion assay
Migration assay
CON
SRGN
ΔGAG
mGAG
KYSE150
KYSE410
E
KYSE30
KYSE150
KYSE410
T.Tn
Relative cell viability
Days
CON
SRGN
ΔGAG
mGAG
F
KYSE410 / KYSE150
CM from KYSE150 with CON, SRGN, ΔGAG, or mGAG overexpression
KYSE150 CM
CM: Medium
CON
SRGN
ΔGAG
mGAG
KYSE150
KYSE410

Figure S4. Comparison of *SRGN, ∆GAG, mGAG* overexpression on invasion and migration of ESCC cells. (A) Design of expression constructs for *SRGN, ∆GAG and mGAG* expression. Wild-type GAG attachment region is indicated in red color. The ∆GAG construct had a truncated GAG attachment region (striped box). The mGAG construct had a mutated GAG attachment region (indicated in blue) with eight serine residues mutated to alanine. The SRGN264 primers were designed to cover a region from exon 2 to the end of GAG attachment region to give a 264 bp PCR product. Primers SRGN167 were designed to amplify the region starting from the exon 1-exon 2 junction to the exon 2-exon 3 junction, which excludes the GAG attachment region of *SRGN*, giving a 167 bp PCR product. (B-C) Validation of *SRGN, ∆GAG, mGAG* overexpression in ESCC cells by (B) RT-PCR, and (C) western blotting. (D) Representative images showing the effects of *SRGN, ∆GAG, mGAG* overexpression on invasive and migration potential of ESCC cells. (E) Effects of *SRGN, ∆GAG, mGAG* overexpression on ESCC cell viability. (F) Experimental scheme (left panel) and representative images of invasion assay (right panel) in which the effects of CM from KYSE150 expressing *SRGN*, *∆GAG*, *mGAG*, and CON were tested as chemoattractant for invasion of KYSE150 and KYSE410 cells. Scale bars, 100 µm.

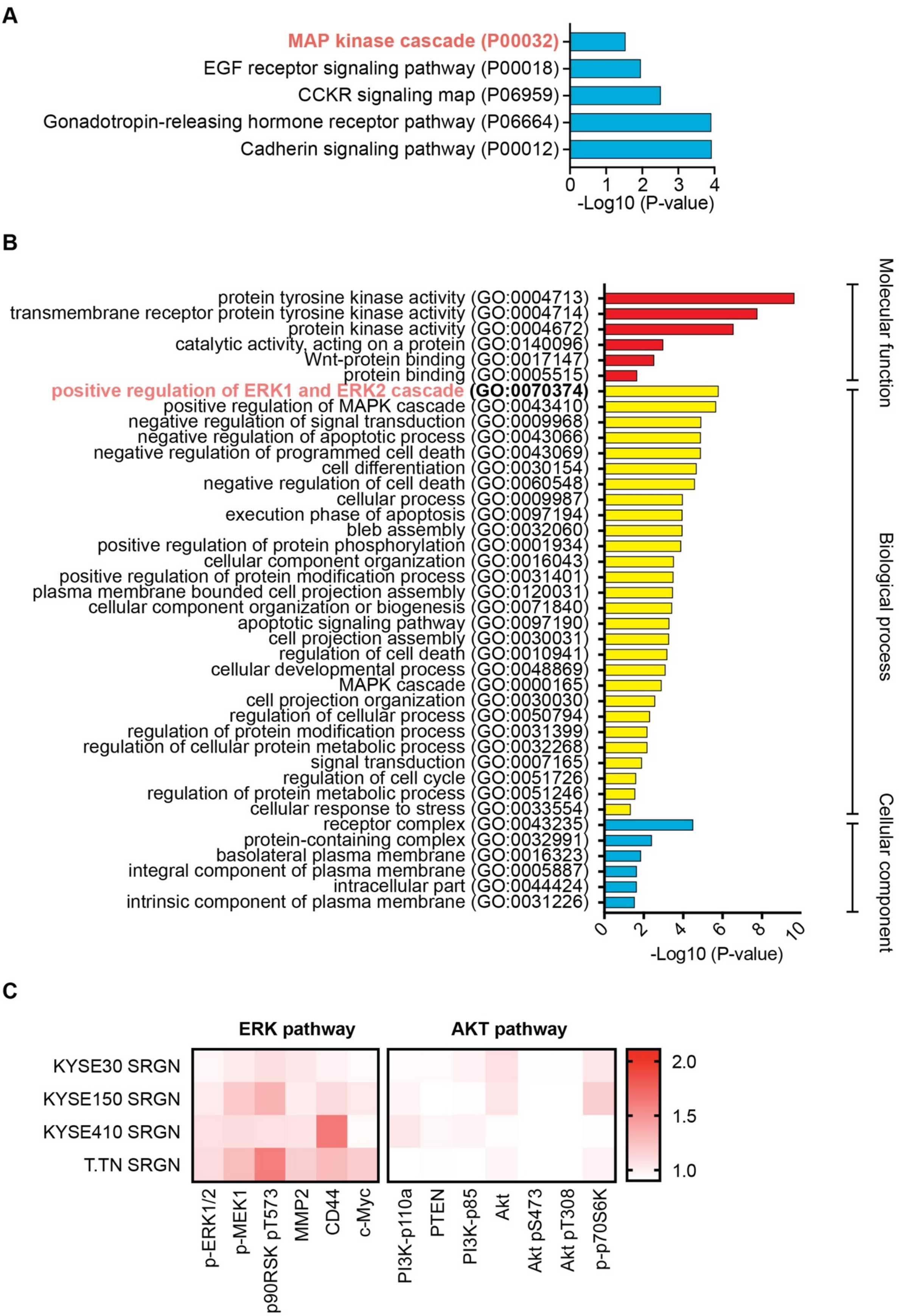

A
MAP kinase cascade (P00032)
EGF receptor signaling pathway (P00018)
CCKR signaling map (P06959)
Gonadotropin-releasing hormone receptor pathway (P06664)
Cadherin signaling pathway (P00012)
0 1 2 3 4
-Log10 (P-value)
B
protein tyrosine kinase activity (GO:0004713)
transmembrane receptor protein tyrosine kinase activity (GO:0004714)
protein kinase activity (GO:0004672)
catalytic activity, acting on a protein (GO:0140096)
Wnt-protein binding (GO:0017147)
protein binding (GO:0005515)
positive regulation of ERK1 and ERK2 cascade (GO:0070374)
positive regulation of MAPK cascade (GO:0043410)
negative regulation of signal transduction (GO:0009968)
negative regulation of apoptotic process (GO:0043066)
negative regulation of programmed cell death (GO:0043069)
cell differentiation (GO:0030154)
negative regulation of cell death (GO:0060548)
cellular process (GO:0009987)
execution phase of apoptosis (GO:0097194)
bleb assembly (GO:0032060)
positive regulation of protein phosphorylation (GO:0001934)
cellular component organization (GO:0016043)
positive regulation of protein modification process (GO:0031401)
plasma membrane bounded cell projection assembly (GO:0120031)
cellular component organization or biogenesis (GO:0071840)
apoptotic signaling pathway (GO:0097190)
cell projection assembly (GO:0030031)
regulation of cell death (GO:0010941)
cellular developmental process (GO:0048869)
MAPK cascade (GO:0000165)
cell projection organization (GO:0030030)
regulation of cellular process (GO:0050794)
regulation of protein modification process (GO:0031399)
regulation of cellular protein metabolic process (GO:0032268)
signal transduction (GO:0007165)
regulation of cell cycle (GO:0051726)
regulation of protein metabolic process (GO:0051246)
cellular response to stress (GO:0033554)
receptor complex (GO:0043235)
protein-containing complex (GO:0032991)
basolateral plasma membrane (GO:0016323)
integral component of plasma membrane (GO:0005887)
intracellular part (GO:0044424)
intrinsic component of plasma membrane (GO:0031226)
0 2 4 6 8 10
-Log10 (P-value)
Molecular function
Biological process
Cellular component
C
ERK pathway
AKT pathway
KYSE30 SRGN
KYSE150 SRGN
KYSE410 SRGN
T.TN SRGN
p-ERK1/2
p-MEK1
p90RSK pT573
MMP2
CD44
c-Myc
PI3K-p110a
PTEN
PI3K-p85
Akt
Akt pS473
Akt pT308
p-p70S6K
2.0
1.5
1.0

Figure S5. PANTHER pathway and GO analyses of RPPA data. (A) The top 15% upregulated proteins in *SRGN*-overexpressing cells were inputted for PANTHER pathway analysis. Analysis was performed online (http://www.geneontology.org), and the MAPK cascade was amongst the top five most altered pathways. (B) GO enrichment analysis showing the significantly ($P < 0.05$) enriched GO terms in molecular function (red), biological process (yellow), and cellular component (blue). (C) Fold changes of ERK-associated and AKT-associated proteins in *SRGN*-overexpressing cells were visualized as heatmaps.

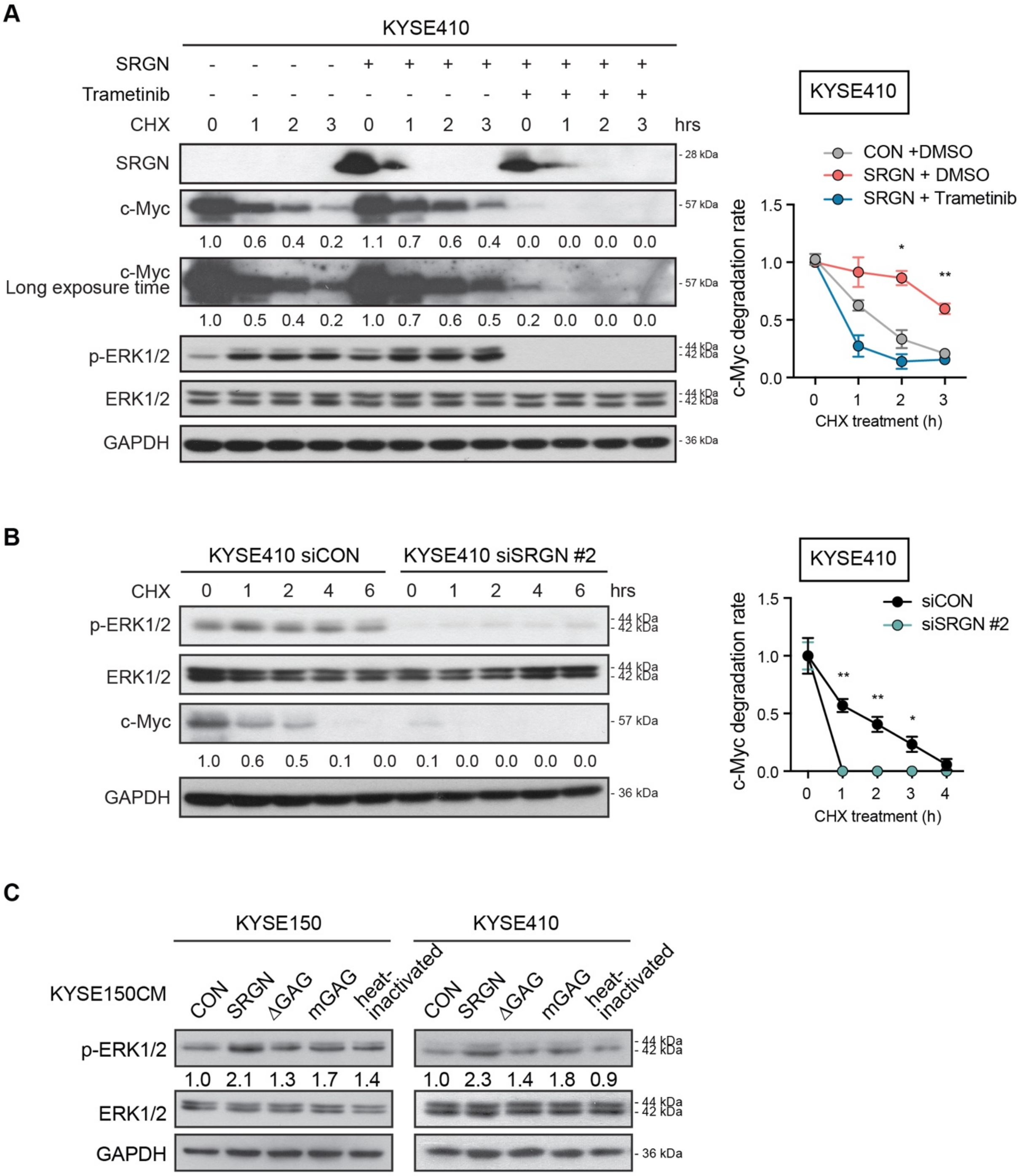
A
KYSE410
SRGN
Trametinib
CHX
hrs
SRGN
c-Myc
1.0 0.6 0.4 0.2 1.1 0.7 0.6 0.4 0.0 0.0 0.0 0.0
c-Myc
Long exposure time
1.0 0.5 0.4 0.2 1.0 0.7 0.6 0.5 0.2 0.0 0.0 0.0
p-ERK1/2
ERK1/2
GAPDH
- 28 kDa
- 57 kDa
- 44 kDa
- 42 kDa
- 36 kDa
KYSE410
CON +DMSO
SRGN + DMSO
SRGN + Trametinib
c-Myc degradation rate
CHX treatment (h)
B
KYSE410 siCON
KYSE410 siSRGN #2
CHX
0 1 2 4 6 0 1 2 4 6 hrs
p-ERK1/2
ERK1/2
c-Myc
1.0 0.6 0.5 0.1 0.0 0.1 0.0 0.0 0.0 0.0
GAPDH
KYSE410
siCON
siSRGN #2
c-Myc degradation rate
CHX treatment (h)
C
KYSE150
KYSE410
KYSE150CM
CON
SRGN
ΔGAG
mGAG
heat-inactivated
p-ERK1/2
1.0 2.1 1.3 1.7 1.4
1.0 2.3 1.4 1.8 0.9
ERK1/2
GAPDH

Figure S6. SRGN stabilizes c-Myc through the ERK pathway and the effect of CM from SRGN-overexpressing cells on activation of ERK pathway. (A) KYSE410-SRGN cells with or without trametinib treatment (100 nM) were incubated in 100 µg/mL CHX for indicated hours before western blotting (left panel). The numbers below the c-Myc blots are the bands intensities that were normalized against GAPDH and then expressed relative to that at 0 hour time point. The relative c-Myc degradation rate is presented in the graph (right panel). (B) KYSE410 cells with *SRGN*-knockdown were subjected to CHX chase assay (left panel) and the band intensities were quantified (right panel). (C) Western blot analysis of ERK phosphorylation status in non-transfected ESCC cells treated with CM of KYSE150 with SRGN-, ΔGAG-, mGAG-expressing cells. Heat-inactivated CM from SRGN-expressing cells was also included for comparison. The numbers below the p-ERK1/2 blots are the signal densities of the p-ERK1/2 bands that were normalized against GAPDH and then expressed numerically relative to the control, which was set to 1.0.

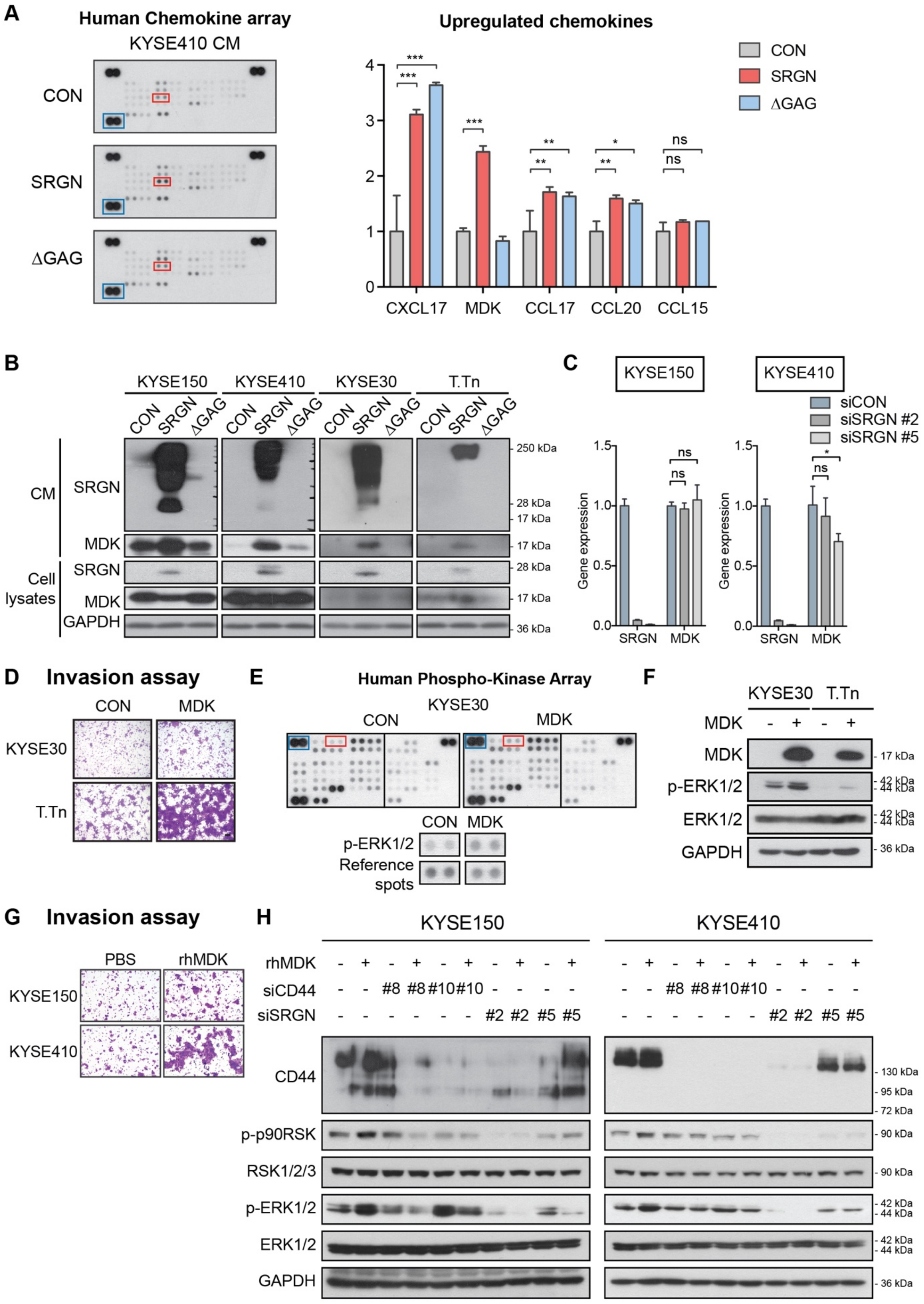
A
Human Chemokine array
KYSE410 CM
CON
SRGN
ΔGAG
Upregulated chemokines
CON
SRGN
ΔGAG
CXCL17
MDK
CCL17
CCL20
CCL15
B
KYSE150
KYSE410
KYSE30
T.Tn
CON SRGN ΔGAG
CM
SRGN
MDK
Cell lysates
SRGN
MDK
GAPDH
250 kDa
28 kDa
17 kDa
36 kDa
C
KYSE150
KYSE410
siCON
siSRGN #2
siSRGN #5
Gene expression
SRGN
MDK
D
Invasion assay
CON
MDK
KYSE30
T.Tn
E
Human Phospho-Kinase Array
KYSE30
CON
MDK
p-ERK1/2
Reference spots
F
KYSE30
T.Tn
MDK
p-ERK1/2
ERK1/2
GAPDH
17 kDa
42 kDa
44 kDa
36 kDa
G
Invasion assay
PBS
rhMDK
KYSE150
KYSE410
H
KYSE150
KYSE410
rhMDK
siCD44
siSRGN
CD44
p-p90RSK
RSK1/2/3
p-ERK1/2
ERK1/2
GAPDH
130 kDa
95 kDa
72 kDa
90 kDa
42 kDa
44 kDa
36 kDa

Figure S7. Effects of SRGN-induced MDK and MDK overexpression on ESCC cell invasion and ERK phosphorylation. (A) Human chemokine profiling of CM of KYSE410 cells expressing vector control (CON), *SRGN* and Δ*GAG*. Red and blue frames mark the spots for MDK and reference, respectively, which were shown in Figure 5A. The right panel shows other chemokines that were increased in the CM from *SRGN*-overexpressing cells. (B) Expression of MDK in cell lysates and CM of four ESCC cell lines with *SRGN* or Δ*GAG* overexpression. (C) Effect of *SRGN* knockdown on mRNA expression of *MDK*. (D) Representative images of invasion assay showing the effect of *MDK* overexpression on invasion of KYSE30 and T.Tn cells. Scale bar, 100 µm. (E) Human phospho-kinase profiling of KYSE30 cells with *MDK* overexpression compared with vector control (upper panel). Red and blue frames mark the spots for p-ERK1/2 and reference, respectively, which were enlarged in the lower panel. (F) Effect of *MDK* overexpression on p-ERK1/2 expression in KYSE30 and T.Tn cells. (G) Representative images of invasion assay showing the effect of rhMDK (500 ng/mL) on invasion of KYSE150 and KYSE410 cells, compared with PBS. Scale bar, 100 µm. (H) Western blotting analysis of the effects of rhMDK treatment on activating ERK pathway in *CD44*-knockdown cells and *SRGN*-knockdown cells. Two siRNAs (siCD44 #8 and siCD44 #10) targeting CD44 and two siRNAs (siSRGN #2 and siSRGN #5) targeting SRGN were transfected into the cells. The duration of rhMDK treatment was 1 hour.

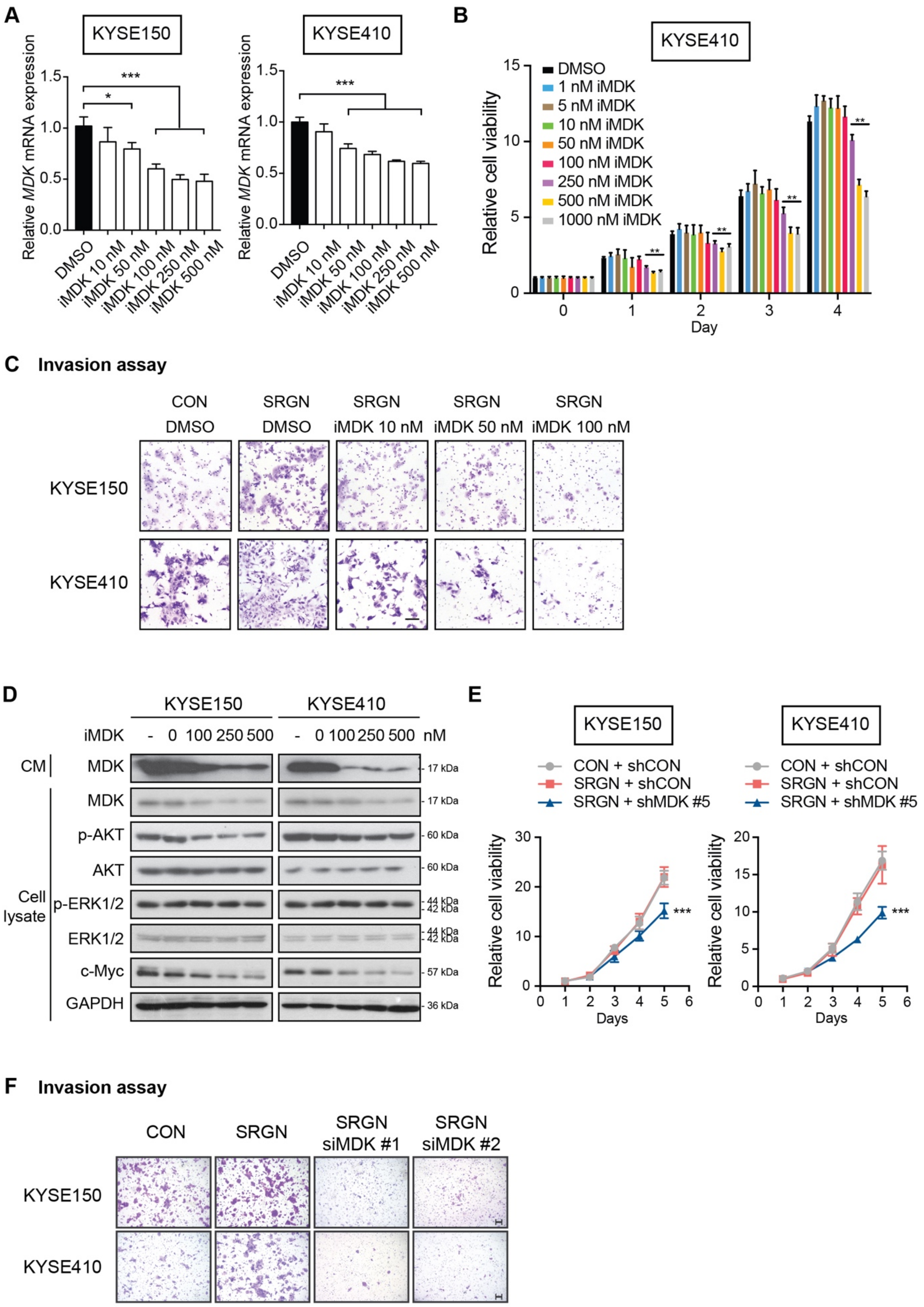

A
KYSE150
Relative MDK mRNA expression
DMSO
iMDK 10 nM
iMDK 50 nM
iMDK 100 nM
iMDK 250 nM
iMDK 500 nM
KYSE410
B
KYSE410
Relative cell viability
DMSO
1 nM iMDK
5 nM iMDK
10 nM iMDK
50 nM iMDK
100 nM iMDK
250 nM iMDK
500 nM iMDK
1000 nM iMDK
Day
C Invasion assay
CON DMSO
SRGN DMSO
SRGN iMDK 10 nM
SRGN iMDK 50 nM
SRGN iMDK 100 nM
KYSE150
KYSE410
D
KYSE150
KYSE410
iMDK - 0 100 250 500 - 0 100 250 500 nM
CM
MDK
Cell lysate
MDK
p-AKT
AKT
p-ERK1/2
ERK1/2
c-Myc
GAPDH
17 kDa
60 kDa
44 kDa
42 kDa
57 kDa
36 kDa
E
KYSE150
KYSE410
CON + shCON
SRGN + shCON
SRGN + shMDK #5
Relative cell viability
Days
F Invasion assay
CON
SRGN
SRGN siMDK #1
SRGN siMDK #2
KYSE150
KYSE410

Figure S8. Treatment with MDK inhibitor (iMDK) or *MDK*-knockdown can reverse the effects of SRGN on ESCC cell viability and invasion. (A) The effect of iMDK at different concentrations on endogenous expression of *MDK* in ESCC cells. (B) Cell viability of KYSE410 treated with iMDK at indicated concentrations. (C) Representative images of invasion assay showing the effect of iMDK (10, 50 and 100 nM) on *SRGN*-overexpressing cells. (D) The effects of iMDK on intracellular and extracellular MDK expression, and on p-ERK1/2 and p-AKT expression in parental ESCC cells. (E) Effect of *MDK*-knockdown on viability of *SRGN*-overexpressing ESCC cells. (F) Representative images of invasion assay showing the effects of *MDK*-knockdown on *SRGN*-overexpressing cells. Scale bar, 100 μm.

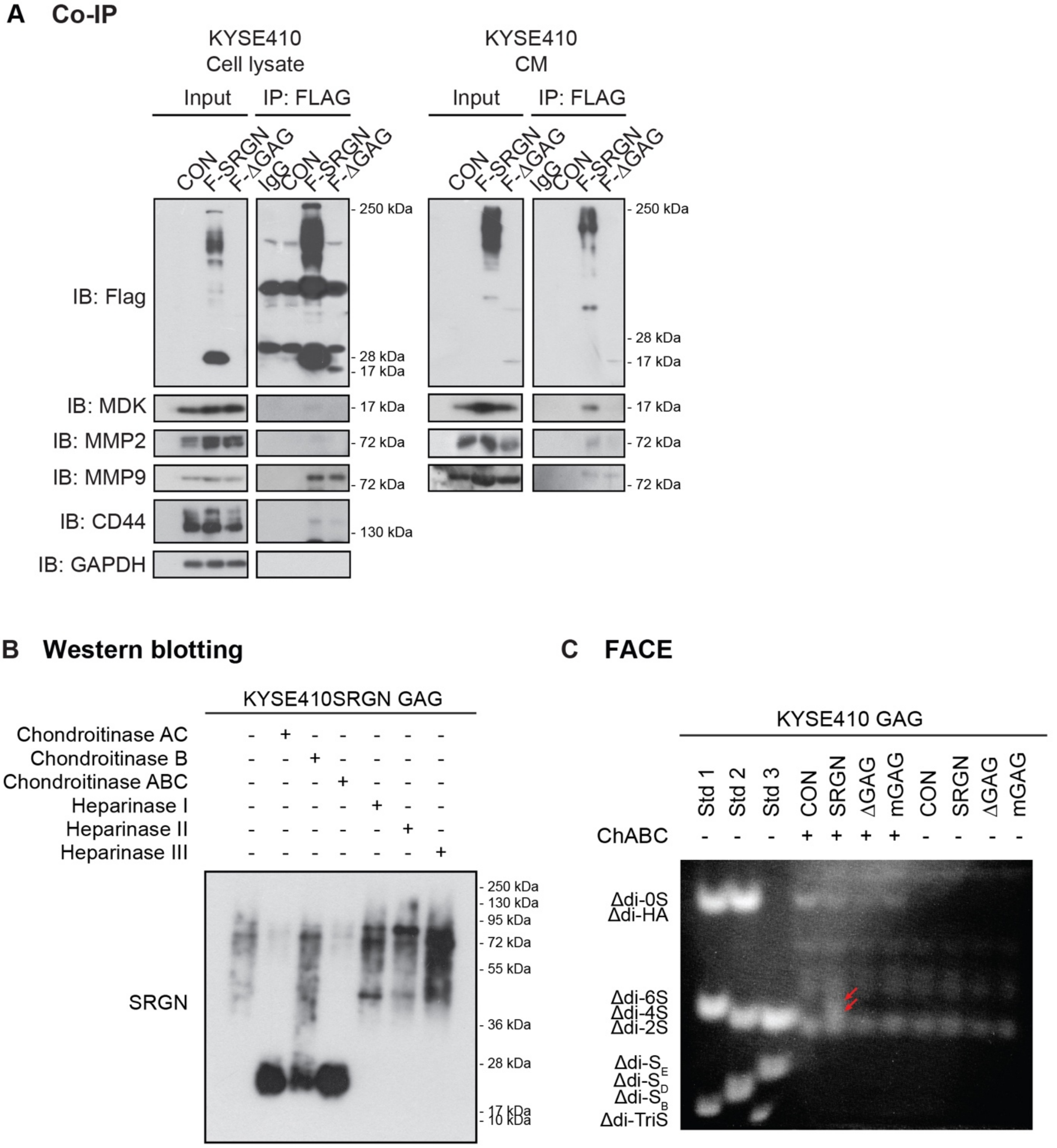

Figure S9. SRGN interacts with MDK, MMP2, MMP9 and CD44. (A) Western blots showing that both the F-SRGN and F-ΔGAG were precipitated using anti-FLAG M2 beads. MMP9 and CD44 were co-precipitated with both F-SRGN and F-ΔGAG. The glycosylated F-SRGN was predominantly precipitated in the CM. MDK and MMP2 were co-precipitated with F-SRGN but not F-ΔGAG. (B) Proteoglycans isolated from CM of KYSE410-SRGN cells were digested with the indicated enzymes before detection of SRGN using western blotting. (C) FACE analysis of disaccharide products in proteoglycan preparations isolated from the CM of KYSE410 cells after chondroitinase ABC treatment. Lanes Std1, Std2, and Std3 contained the standard markers. Red arrows indicate Δdi-4S and Δdi-6S.

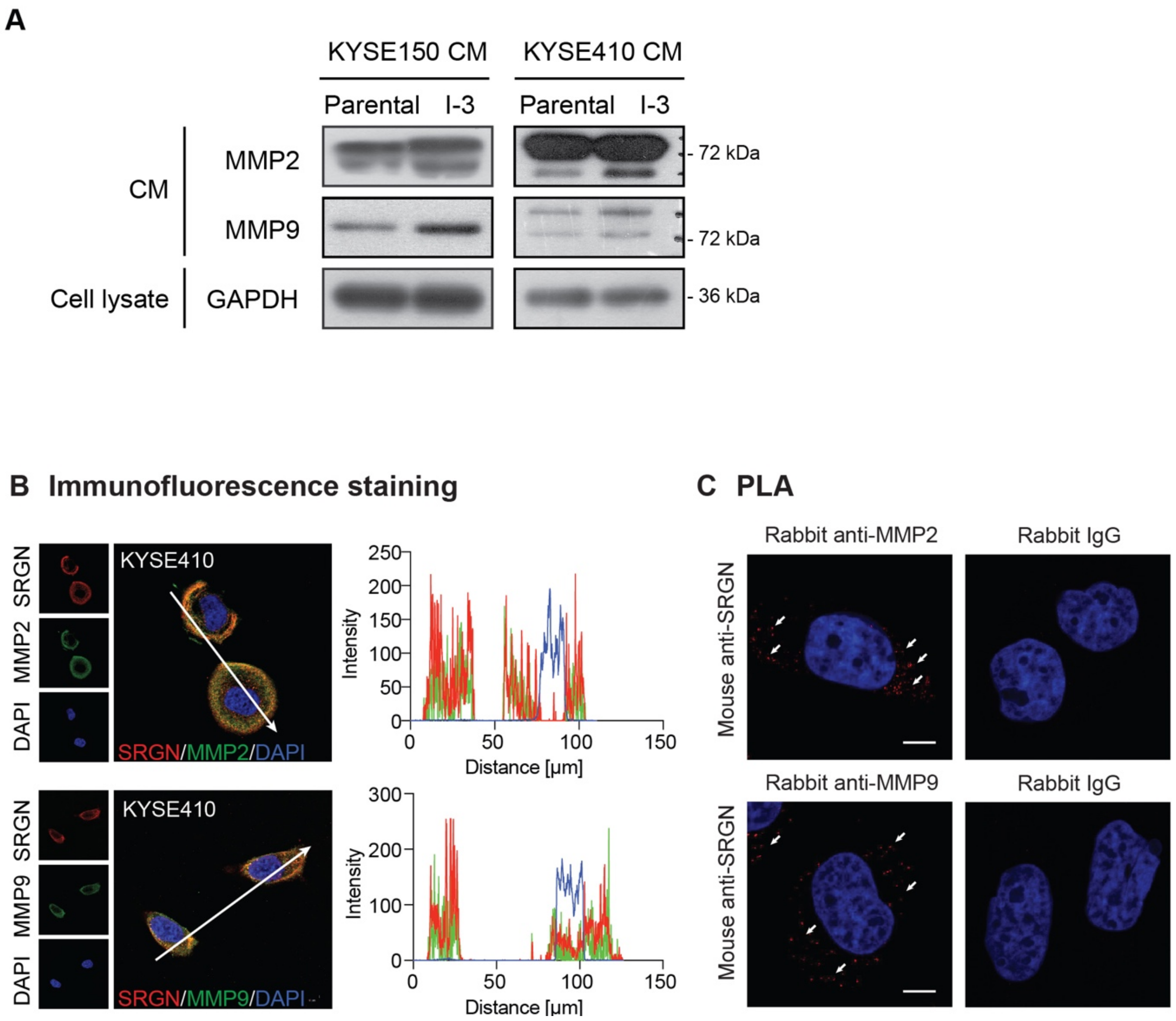

Figure S10. SRGN binds to MMP2 or MMP9. (A) Expression of MMP2 and MMP9 in CM from I-3 cells compared with that from parental cells. GAPDH in the cell lysates was used as loading control. (B) Immunofluorescence staining and (C) PLA showed co-localization and interaction of SRGN with MMP2 and MMP9 in KYSE410 cells. Negative control was conducted by replacing MDK antibody with rabbit IgG. Scale bar, 10 μm.
